# Examining Sex-Differentiated Genetic Effects Across Neuropsychiatric and Behavioral Traits

**DOI:** 10.1016/j.biopsych.2020.12.024

**Published:** 2021-06-15

**Authors:** Joanna Martin, Ekaterina A. Khramtsova, Slavina B. Goleva, Gabriëlla A.M. Blokland, Michela Traglia, Raymond K. Walters, Christopher Hübel, Jonathan R.I. Coleman, Gerome Breen, Anders D. Børglum, Ditte Demontis, Jakob Grove, Thomas Werge, Janita Bralten, Cynthia M. Bulik, Phil H. Lee, Carol A. Mathews, Roseann E. Peterson, Stacey J. Winham, Naomi Wray, Howard J. Edenberg, Wei Guo, Yin Yao, Benjamin M. Neale, Stephen V. Faraone, Tracey L. Petryshen, Lauren A. Weiss, Laramie E. Duncan, Jill M. Goldstein, Jordan W. Smoller, Barbara E. Stranger, Lea K. Davis, Sex Differences Cross-Disorder Analysis Group of the Psychiatric Genomics Consortium, Martin Alda, Marco Bortolato, Christie L. Burton, Enda Byrne, Caitlin E. Carey, Lauren Erdman, Laura M. Huckins, Manuel Mattheisen, Elise Robinson, Eli Stahl

**Affiliations:** a MRC Centre for Neuropsychiatric Genetics and Genomics, Division of Psychological Medicine and Clinical Neurosciences, Cardiff University, Cardiff, United Kingdom; b Social, Genetic and Developmental Psychiatry Centre, Institute of Psychiatry, Psychology & Neuroscience, King’s College London, London, United Kingdom; c National Institute for Health Research Maudsley Biomedical Research Centre, South London and Maudsley National Health Service Trust, London, United Kingdom; d Section of Genetic Medicine, Department of Medicine and Institute for Genomics and Systems Biology, University of Chicago, Chicago, Illinois; e Center for Genetic Medicine, Department of Pharmacology, Northwestern University, Chicago, Illinois; f Computational Sciences, Janssen Pharmaceuticals, Spring House, Pennsylvania; g Department of Molecular Physiology and Biophysics, Vanderbilt University Medical Center, Nashville, Tennessee; h Division of Genetic Medicine, Department of Medicine, Vanderbilt University Medical Center, Nashville, Tennessee; i Department of Psychiatry and Behavioral Sciences, Vanderbilt University Medical Center, Nashville, Tennessee; j Psychiatric & Neurodevelopmental Genetics Unit, Massachusetts General Hospital, Harvard Medical School, Boston, Massachusetts; k Department of Psychiatry, Massachusetts General Hospital, Harvard Medical School, Boston, Massachusetts; l Center for Genomic Medicine, Massachusetts General Hospital, Harvard Medical School, Boston, Massachusetts; m Analytic and Translational Genetics Unit, Department of Medicine, Massachusetts General Hospital, Harvard Medical School, Boston, Massachusetts; n Department of Obstetrics and Gynecology, Massachusetts General Hospital, Harvard Medical School, Boston, Massachusetts; o Stanley Center for Psychiatric Research, Broad Institute of MIT and Harvard, Cambridge, Massachusetts; p Department of Psychiatry, University of California San Francisco, San Francisco, California; q Institute for Human Genetics, University of California San Francisco, San Francisco, California; r Weill Institute for Neurosciences, University of California San Francisco, San Francisco, California; s Department of Psychiatry and Behavioral Sciences, Stanford University, Stanford, California; t Department of Psychiatry, University of North Carolina at Chapel Hill, Chapel Hill, North Carolina; u Department of Nutrition, University of North Carolina at Chapel Hill, Chapel Hill, North Carolina; v Department of Psychiatry, University of Florida, Gainesville, Florida; w Genetics Institute, University of Florida, Gainesville, Florida; x Department of Psychiatry, Virginia Commonwealth University, Richmond, Virginia; y Virginia Institute for Psychiatric and Behavioral Genetics, Virginia Commonwealth University, Richmond, Virginia; z Department of Health Sciences Research, Division of Biomedical Statistics and Informatics, Mayo Clinic, Rochester, Minnesota; aa Department of Biochemistry and Molecular Biology, Indiana University School of Medicine, Indianapolis, Indiana; bb Genetic Epidemiology Research Branch, National Institute of Mental Health, National Institutes of Health, Bethesda, Maryland; cc Department of Psychiatry, State University of New York Upstate Medical University, Syracuse, New York; dd Department of Neuroscience and Physiology, State University of New York Upstate Medical University, Syracuse, New York; ee Department of Psychiatry and Neuropsychology, School for Mental Health and Neuroscience, Faculty of Health, Medicine, and Life Sciences, Maastricht University, Maastricht, The Netherlands; ff Department of Human Genetics, Radboud University Medical Center, Nijmegen, The Netherlands; gg Department of Medical Epidemiology and Biostatistics, Karolinska Institutet, Stockholm, Sweden; hh The Lundbeck Foundation Initiative for Integrative Psychiatric Research, iPSYCH, Aarhus, Denmark; ii Department of Biomedicine, Aarhus University, Aarhus, Denmark; jj Center for Genomics and Personalized Medicine, Aarhus, Denmark; kk Institute of Biological Psychiatry, Mental Health Center Sct. Hans, Mental Health Services Copenhagen, Copenhagen, Denmark; ll Department of Clinical Medicine, Faculty of Health, University of Copenhagen, Copenhagen, Denmark; mm Section for GeoGenetics, GLOBE Institute, University of Copenhagen, Copenhagen, Denmark; nn The Lundbeck Foundation Initiative for Integrative Psychiatric Research (iPSYCH), Copenhagen, Denmark; oo Institute for Molecular Bioscience, University of Queensland, Brisbane, Australia; pp School of Life Sciences, Fudan University, Shanghai, China

**Keywords:** Behavioral, Genetic correlation, GWAS, Heritability, Psychiatric, Sex differences

## Abstract

**Background:**

The origin of sex differences in prevalence and presentation of neuropsychiatric and behavioral traits is largely unknown. Given established genetic contributions and correlations, we tested for a sex-differentiated genetic architecture within and between traits.

**Methods:**

Using European ancestry genome-wide association summary statistics for 20 neuropsychiatric and behavioral traits, we tested for sex differences in single nucleotide polymorphism (SNP)-based heritability and genetic correlation (*r*_*g*_ < 1). For each trait, we computed per-SNP *z* scores from sex-stratified regression coefficients and identified genes with sex-differentiated effects using a gene-based approach. We calculated correlation coefficients between *z* scores to test for shared sex-differentiated effects. Finally, we tested for sex differences in across-trait genetic correlations.

**Results:**

We observed no consistent sex differences in SNP-based heritability. Between-sex, within-trait genetic correlations were high, although <1 for educational attainment and risk-taking behavior. We identified 4 genes with significant sex-differentiated effects across 3 traits. Several trait pairs shared sex-differentiated effects. The top genes with sex-differentiated effects were enriched for multiple gene sets, including neuron- and synapse-related sets. Most between-trait genetic correlation estimates were not significantly different between sexes, with exceptions (educational attainment and risk-taking behavior).

**Conclusions:**

Sex differences in the common autosomal genetic architecture of neuropsychiatric and behavioral phenotypes are small and polygenic and unlikely to fully account for observed sex-differentiated attributes. Larger sample sizes are needed to identify sex-differentiated effects for most traits. For well-powered studies, we identified genes with sex-differentiated effects that were enriched for neuron-related and other biological functions. This work motivates further investigation of genetic and environmental influences on sex differences.

SEE COMMENTARY ON PAGE e63

Despite widespread evidence of sex differences across human complex traits, including neuropsychiatric and behavioral phenotypes (1), the etiology of these differences remains poorly understood. Accumulating evidence suggests that sex differences in complex human phenotypes are likely to include an autosomal genetic component beyond that contributed by sex chromosomes (2, 3, 4, 5). Understanding the biological basis of sex differences in human disease, including neuropsychiatric phenotypes, is critical for developing sex-informed diagnostics and therapeutics and realizing the promise of precision medicine (4). Moreover, genetic variants with sex-differentiated effects across multiple traits may influence patterns of comorbidity for neuropsychiatric and related behavioral phenotypes, suggesting the need for cross-disorder genetic analyses to be evaluated in the context of sex-differentiated effects (6, 7, 8, 9, 10, 11).

Neuropsychiatric and behavioral phenotypes are generally characterized by a complex and highly polygenic etiology (12). Many of these traits share common genetic risk variants (13,14). Specific genetic loci with pleiotropic effects are known to impact risk for multiple related phenotypes (12). However, it is not yet known whether these pleiotropic effects are consistent across sex.

Several recent studies have investigated sex-differentiated genetic effects for a number of neuropsychiatric traits (15, 16, 17, 18, 19, 20, 21, 22, 23, 24, 25, 26, 27, 28, 29). Given evidence of phenotypic sex differences in prevalence and presentation as well as genetic correlations between these traits (13), we aimed to systematically test the hypothesis that neuropsychiatric and behavioral phenotypes have a partially sex-differentiated autosomal genetic architecture that may be shared across traits. In this study, we have characterized the 1) sex-dependent genetic architecture for a range of neuropsychiatric and behavioral traits, 2) degree of shared genetic architecture between males and females within each phenotype, and 3) sex-specific patterns of genetic effects shared across traits.

## Methods and Materials

### Datasets

We collected sex-stratified genome-wide association study (GWAS) meta-analysis summary statistics for 20 neuropsychiatric and behavioral traits (Table 1; see Sex-Stratified Datasets in Supplement 1), chosen based on data availability. See Table S1 in Supplement 2 for information about data availability. We used a broad definition of brain-based human complex traits, given the overwhelming evidence of shared genetic effects across such traits (13). We used results from European ancestry GWASs only to minimize any bias that may arise from ancestry differences and because large sex-stratified GWAS summary statistics from other ancestries are not currently available. We analyzed autosomal-only common variants with a minor allele frequency >1%.

**Table 1:** Summary of Analyzed Datasets of Neuropsychiatric and Behavioral Traits

Phenotype (Full Name)	Acronym	Female Cases (*n*)	Female Controls (*n*)	Male Cases (*n*)	Male Controls (*n*)	M:F Case Ratio	Sample Type	Consortium/Group	Reference
Attention-Deficit/Hyperactivity Disorder	ADHD	4945	16,246	14,154	17,948	2.86	Clinical case-control	PGC+iPSYCH	(15)
Alcohol Dependence	ALCD	2504	6033	5932	9412	2.37	Clinical case-control	PGC	(16)
Anxiety Disorders	ANX	3148	191,005	1813	165,175	0.58	General population (UK)	Neale laboratory	(17)
Autism Spectrum Disorder	ASD	7498	24,309	30,168	32,417	4.02	Clinical case-control	PGC+iPSYCH	(18,19)
Bipolar Disorder	BD	10,753	14,225	7331	13,572	0.68	Clinical case-control	PGC2	(20)
Cannabis Use (Ever)	CUE	17,244	71,742	17,414	50,737	1.01	General population (UK)	N/A	N/A
Insomnia	INS	19,521	39,846	12,863	40,776	0.66	General population (UK)	N/A	(21)
Major Depressive Disorder	MDD	10,711	11,745	5021	11,226	0.47	Clinical and population case-control	PGC1	(20)
Major Depressive Disorder	N/A^a^	13,492	180,661	7156	159,832	0.53	General population (UK)	Neale laboratory	(17)
Major Depressive Disorder Recurrent	MDDR	6026	8949	2643	8162	0.44	Clinical case-control	PGC1	(20)
Obsessive-Compulsive Disorder	OCD	1525	4307	1249	2789	0.82	Clinical case-control	PGC	(22)
Posttraumatic Stress Disorder	PTSD	968	2457	585	4025	0.60	Clinical case-control	PGC	(23)
Risk-Taking Behavior	RTB	32,285	143,678	51,392	100,984	1.59	General population (UK)	N/A	(24)
Schizophrenia	SCZ	9837	16,763	18,346	17,122	1.86	Clinical case-control	PGC2	(20)
Smoking (Current)	SMKC	16,995	176,392	20,093	146,226	1.18	General population (UK)	Neale laboratory	(17)
Smoking (Previous)	SMKP	62,305	131,082	65,245	101,074	1.05	General population (UK)	Neale laboratory	(17)

		Females (*n*)		Males (*n*)					

Alcohol Use	ALCC	59,088		53,088		0.90	General population (UK)		(25)
Alcohol Use	N/A^a^	85,800		55,120		0.64	General population		(26)
Age at First Birth	AFB	189,656		48,408		0.26	General population		(27)
Educational Attainment	EA	182,286		146,631		0.80	General population		(28)
Number of Children Ever Born	NEB	225,230		103,909		0.46	General population		(27)
Neuroticism	NEU	144,660		142,875		0.99	General population (UK)		(29)

F, female; M, male; N/A, not applicable; PGC, Psychiatric Genomics Consortium; UK, United Kingdom.

a These summary statistics were not used for analysis (see Sex-Stratified Datasets in Supplement 1 for details).

### Sex-Specific Single Nucleotide Polymorphism–Based Heritability

For each trait, we calculated sex-specific observed scale single nucleotide polymorphism (SNP)-based heritability (SNP-*h*^*2*^) using linkage disequilibrium (LD) score regression (LDSC) with precomputed European ancestry LD scores (excluding SNPs in the HLA/MHC [human leukocyte antigen/major histocompatibility complex] region; chr6:25-34M) (30). For 11 binary traits, we also estimated liability scale SNP-*h*^*2*^, using sex-specific population prevalence rates from two sources, as described below. For comparison with this primary analysis, we also used a second method, LDAK-SumHer (31), to estimate SNP-*h*^*2*^, using the LD-adjusted kinships (LDAK) heritability model.

We obtained sex-specific trait prevalence estimates from the United States (32) and cumulative incidence rates from Denmark (33) to compare the SNP-*h*^*2*^ estimates using two different sources of information. See Sex-Specific Trait Prevalences for Estimating SNP-h2 in Supplement 1 and Tables S2 and S3 in Supplement 2 for details.

For traits with nonzero SNP-*h*^*2*^ estimates (i.e., where confidence intervals did not overlap with zero) in both sexes, we tested whether sex-specific SNP-*h*^*2*^ estimates were significantly different by calculating *z* scores using equation 1 (below) and obtaining corresponding *p* values from a normal distribution. We corrected for multiple tests using Bonferroni (*n* = 12 independent tests for *n* = 5 continuous traits and *n* = 7 binary traits with nonzero liability scale SNP-*h*^*2*^ in both sexes; *p* = .0042).

(1) $$z\;-\;score\;=\;\frac{STAT_{female}-STAT_{male}}{\sqrt{SE_{female}^{2}+SE_{male}^{2}}}$$

In equation 1, STAT can be any statistic for which we want to assess the difference between the sexes, including SNP-*h*^*2*^, *r_g_*, and GWAS β values; SE is the standard error for the statistic. This test is well calibrated when STAT/SE is normally distributed and the test statistics are independent between sexes and is conservative if the statistics are positively correlated.

### Genetic Correlation

**Figure 1:**
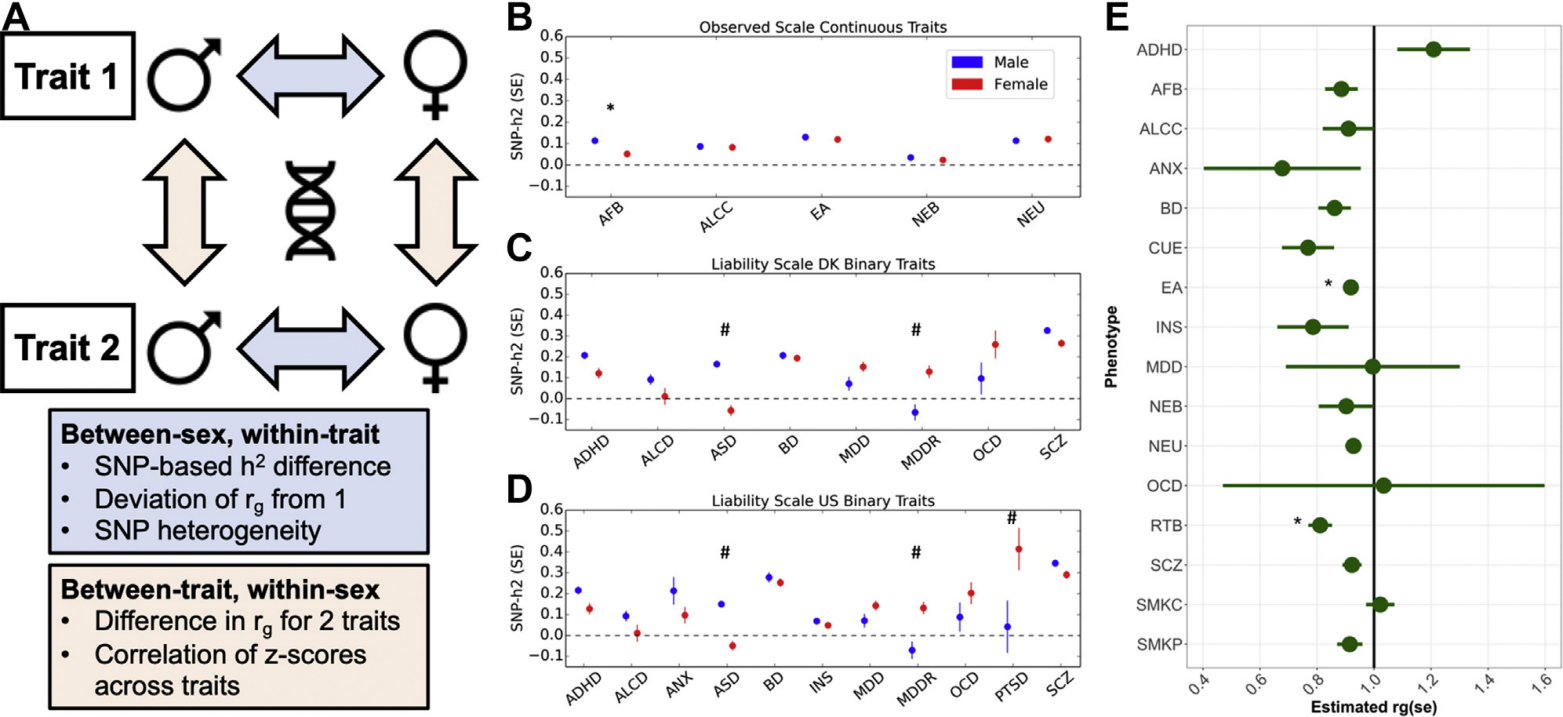
**(A)** Schematic illustration of the key analyses used to investigate between-sex, within-trait and between-trait, within-sex differences. **(B–D)** Estimates of sex stratified SNP-based heritability (SNP-*h*^*2*^) on **(B)** the observed scale for continuous traits and the liability scale using population prevalence based on **(C)** Denmark (DK) and **(D)** the United States (US). Estimates were obtained from linkage disequilibrium score regression. Points represent the estimated SNP-*h*^*2*^ in males (blue) and females (red), while bars represent SE of the SNP-*h*^*2*^ estimates. Significant sex difference in heritability is denoted as follows: ∗*p* < .0042 (adjusted *p* value threshold corrected for multiple testing using Bonferroni). #Traits for which significance in difference is not interpretable owing to negative or nonsignificant from zero SNP-*h*^*2*^ value for one of the measurements. **(E)** Within-trait, between-sex genetic correlation (*r*_*g*_) estimates using linkage disequilibrium score regression. Points represent the estimated *r*_*g*_, and bars represent SE of the *r*_*g*_ estimates. Significant deviation from 1 is denoted as follows: ∗*p* < .0031 (adjusted *p* value threshold corrected for multiple testing using Bonferroni). ADHD, attention-deficit/hyperactivity disorder; AFB, age at first birth; ALCC, alcohol use; ALCD, alcohol dependence; ANX, anxiety disorders; ASD, autism spectrum disorder; BD, bipolar disorder; CUE, cannabis use (ever); EA, educational attainment; INS, insomnia; MDD, major depressive disorder; MDDR, major depressive disorder recurrent; NEB, number of children ever born; NEU, neuroticism; OCD, obsessive-compulsive disorder; PTSD, posttraumatic stress disorder; RTB, risk-taking behavior; SCZ, schizophrenia; SMKC, smoking (current); SMKP, smoking (previous); SNP, single nucleotide polymorphism.

We used LDSC to estimate genetic correlations (*r_g_*) 1) between sexes, within each trait, and 2) between each trait pair, within sex (Figure 1A). For between-sex, within-trait correlations, we tested the null hypothesis that *r*_*g*_ < 1 using a 1-tailed test compared with a normal distribution (*z* = (1 − *r*_*g*_)/SE). We applied a Bonferroni multiple-testing correction (*p* < .0031 based on 16 traits). Next, we tested whether the between-trait *r_g_* estimates were different for males (*r*_*gM*_) and females (*r*_*gF*_) by using a *z* score approximation based on block jackknife to estimate the standard error of *r*_*gM*_ − *r*_*gF*_ in LDSC. As with other LDSC analyses, this approach is robust to sample overlap. We applied a false discovery rate multiple-testing correction.

### Between-Sex, Within-Trait Genetic Heterogeneity

Given that only summary statistics from sex-stratified GWASs were available, the analysis of sex-differentiated genetic effects was limited to the following *z* score approach. For each SNP in the sex-stratified GWAS of each trait, we assessed between-sex, within-trait heterogeneity using *z* scores (which are correlated with Cochran’s *Q* statistic but provide directionality of the effect) as in equation 1. This test quantifies the sex difference in SNP association effect size, similar to, although not the same as, an interaction test (34).

### Sharing of Variants With Sex-Differentiated Effects Across Traits

To assess which traits share sex-differentiated effects (i.e., variants at the extreme ends of the *z* score distribution), we assessed the Pearson correlation coefficient between *z* scores (i.e., the differences of β values from male-only and female-only GWASs) for pairs of traits. Given that there are many nonindependent observations, owing to SNPs in LD, we used a block jackknife approach to estimate the significance of the Pearson correlation (35,36). SNPs were assigned to 1 of 1000 contiguous blocks based on genomic position. For each trait pair, Pearson’s correlation was calculated on the full set of *z* scores and then recalculated after each block was removed, thus estimating the jackknife error and *p* values.

### Gene-Based Analysis, Differential Gene Expression, and Gene-Set Enrichment Analysis of Genes With Sex-Differentiated Effects

We used the Functional Mapping and Annotation of GWAS (FUMA) SNP2GENE web tool (37) to perform gene-based analysis using MAGMA v1.08 (38,39). We examined whether the genes exhibiting a genome-wide significant sex difference (from MAGMA) demonstrate sex-differentiated gene expression in brain tissues from the Genotype-Tissue Expression project v8 (https://www.gtexportal.org/home/datasets) (20). After mapping SNPs to genes (using a default window size of 0), we performed gene set enrichment analysis on the union (across phenotypes) of genes with sex-differentiated effects using GSEA (https://software.broadinstitute.org/gsea/index.jsp). See Gene-Based Analysis, Differential Gene Expression, and Gene-Set Enrichment Analysis of Genes With Sex-Differentiated Effects in Supplement 1 for details.

## Results

### Sex-Stratified SNP-*h*^*2*^ Estimates

Sex-specific SNP-*h*^*2*^ estimates using LDSC are presented in Figure 1B–D, with details provided in Table S4 in Supplement 2. Several traits (posttraumatic stress disorder and recurrent major depressive disorder [MDD] in males and autism spectrum disorder (ASD) and alcohol dependence in females) did not have sufficient power (or had excessive heterogeneity) and we did not detect a polygenic signal, and therefore sex differences could not be assessed. Thus, although we report sex difference estimates for all traits in Table S4 in Supplement 2, these cannot be reliably interpreted for these 4 traits, as one of the sexes exhibited a near-zero SNP-*h*^*2*^ estimate. The liability scale SNP-*h*^*2*^ estimates using population prevalence from the United States and cumulative incidence from Denmark were highly correlated (*r* = .97, *p* = 4.7 × 10^−10^) (Figure S1 in Supplement 1). Age at first birth was the only trait with a significant (after multiple testing correction; *p* < .0042) sex difference in SNP-*h*^*2*^ estimates (females: SNP-*h*^*2*^ = 0.052, SE = 0.004; males: SNP-*h*^*2*^ = 0.113, SE = 0.010; *z* score = −5.81, *p* = 6.43 × 10^−9^).

Observed scale SNP-*h*^*2*^ estimates based on LDAK-SumHer were somewhat higher than the estimates obtained in LDSC and moderately correlated with them (*r* = .69, *p* = 8.5 × 10^−7^ for all traits; *r* = .85, *p* = 3.3 × 10^−11^ excluding the 4 traits for which SNP-*h*^*2*^ could not be reliably estimated in LDSC); see Table S5 in Supplement 2 and Figures S1 and S2 in Supplement 1 for details. Higher estimates from the LDAK model relative to the LDSC model have been previously observed (31,38). In contrast to LDSC results, age at first birth did not show a significant sex difference (*z* score = 1.94, *p* = .052), with an effect in the opposite direction to that observed using LDSC. Using LDAK, the liability scale (adjusted based on each population) SNP-*h*^*2*^ estimates differed by sex for the following traits: recurrent MDD (United States: *z* score = −4.68, *p* = 2.84 × 10^−6^; Denmark: *z* score = −4.46, *p* = 8.06 × 10^−6^), ASD (United States: *z* score = 2.94, *p* = .0033; Denmark: *z* score = 3.28, *p* = .0011), and schizophrenia (Denmark: *z* score = −3.16, *p* = .0016). These results were not observed using LDSC, and indeed SNP-*h*^*2*^ could not be estimated reliably in LDSC for ASD in females or recurrent MDD in males. The biggest discrepancies between estimates obtained from LDSC and LDAK were for the traits with the smallest sample sizes (Figure S3 in Supplement 1). The SNP-*h*^*2*^ results for attention-deficit/hyperactivity disorder (ADHD) and ASD were similar, albeit somewhat higher, for both LDSC and LDAK when using estimates based on a Danish child-specific study (39) compared with using prevalence estimates from the whole Danish population (Tables S4 and S5 in Supplement 2) (33).

### Between-Sex, Within-Trait Genetic Correlation Analysis

We quantified the genetic correlation between males and females for each trait (excluding the 4 traits where SNP-*h*^*2*^ could not be estimated in one of the sexes) (Figure 1E and Table S6 in Supplement 2). We found moderate-to-high genetic correlations for all traits (*r*_*g*_ = 0.68–1.21); these all differed significantly from zero, and we also detected a significant difference from 1 for risk-taking behavior (*r*_*g*_ = 0.81, SE = 0.04) and educational attainment (*r*_*g*_ = 0.92, SE = 0.02), after correcting for multiple tests (*p* < .0031), suggesting a modest degree of common variant heterogeneity in males and females for these phenotypes.

### Between-Sex, Within-Trait Heterogeneity Across Variants

**Figure 2:**
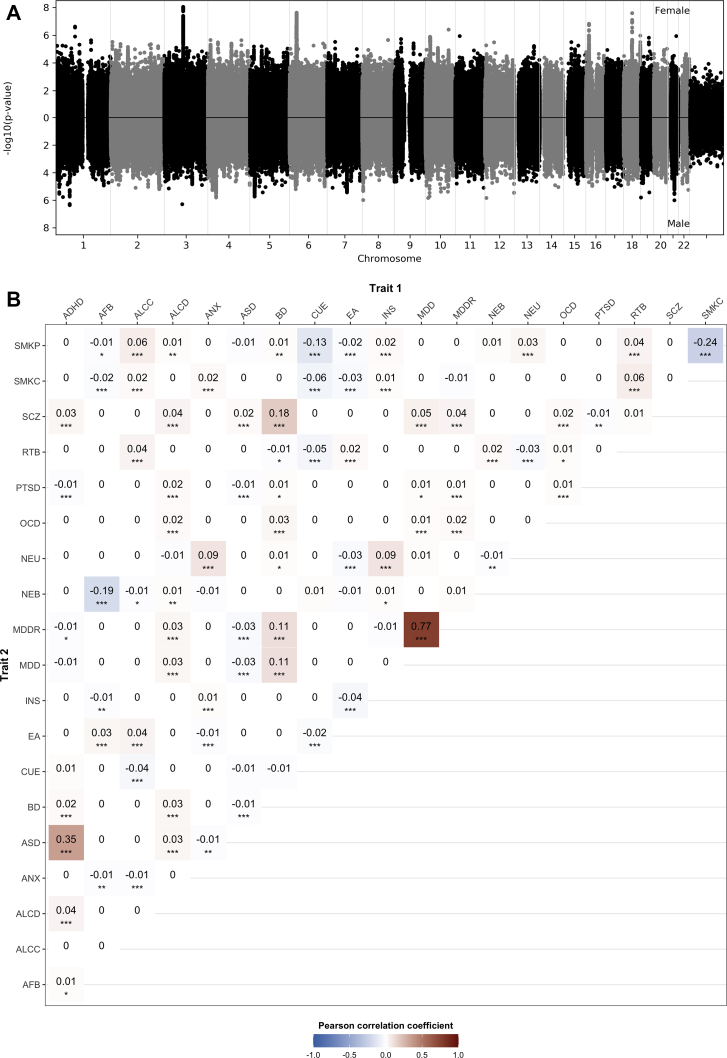
Sharing of variants with sex-differentiated effects between traits. **(A)** Miami plot for female-only (top) and male-only (bottom) genome-wide association studies for cannabis use (ever): female cases: *N* = 17,244; male cases: *N* = 17,414. For each single nucleotide polymorphism, we computed *z* scores using Equation 1. **(B)** Matrix of the Pearson correlation coefficients for pairs of traits. We performed Pearson’s correlation of *z* scores and a block jackknife approach to estimate the significance of the correlation for all pairs of traits. The estimated significance of the coefficients is denoted as follows: ∗*p* < .05, ∗∗*p* < .01, ∗∗∗*p* < .001. Color coding represents positive (red) or negative (blue) correlation. ADHD, attention-deficit/hyperactivity disorder; AFB, age at first birth; ALCC, alcohol use; ALCD, alcohol dependence; ANX, anxiety disorders; ASD, autism spectrum disorder; BD, bipolar disorder; CUE, cannabis use (ever); EA, educational attainment; INS, insomnia; MDD, major depressive disorder; MDDR, major depressive disorder recurrent; NEB, number of children ever born; NEU, neuroticism; OCD, obsessive-compulsive disorder; PTSD, posttraumatic stress disorder; RTB, risk-taking behavior; SCZ, schizophrenia; SMKC, smoking (current); SMKP, smoking (previous).

To assess sex differences in genetic effects of individual common variants, for each trait we computed *z* scores and corresponding *p* values for each SNP, using equation 1. Figure S4 in Supplement 1 shows the quantile-quantile plots of the *z* score *p* values for all traits. While there were no genome-wide significant (*p* < 5 × 10^−8^) differences between male and female β values for any individual SNP, we observed deviation from the expected null distribution (Figure S4 in Supplement 1) for ADHD, lifetime cannabis use, MDD, number of children born, and schizophrenia. Figure 2A shows a Miami plot for female-only (top) and male-only (bottom) lifetime cannabis use GWASs, where we observed several associations that are stronger in females (e.g., chromosomes 3, 6, 16, and 18). As cohorts for lifetime cannabis use are of very similar size, the power to detect association in both sexes is similar.

A gene-based analysis in MAGMA revealed several traits with significant sex-differentiated effects. Gene-based analysis Manhattan plots are shown in Figure S5 in Supplement 1. Traits with significant gene associations include number of children born (*GLB1L2*), risk-taking behavior (*HFE2* and *AGO2*), and schizophrenia (*SLTM*). *SLTM*, which is highly expressed in cerebellum (Genotype-Tissue Expression Portal, www.gtexportal.org), was also identified in a larger (and therefore better-powered) gene-based gene-by-sex interaction for schizophrenia and across schizophrenia, bipolar disorder (BD), and MDD (20). The full set of gene-based MAGMA association statistics is provided in Table S7 in Supplement 3. None of these 4 genes showing differential sex association with the traits shows a significant differential gene expression in the brain tissues from the Genotype-Tissue Expression project v8 (Table S8 in Supplement 2).

### Shared Sex-Differentiated Effects Across Traits

Many psychiatric traits are frequently comorbid and genetically correlated (13); thus, we hypothesized that sex differences in genetic effects might be a property of the SNP or gene, in which case we would expect that the sex difference observed at an SNP or gene would be observed across multiple traits. To test this hypothesis, for each pair of traits, we calculated the Pearson correlation coefficient between the SNP-based *z* scores (i.e., scores reflecting sex-differentiated effects). Figure 2B shows a correlation matrix for pairs of traits. We observed small-to-moderate, but significant, correlations of *z* scores for several trait pairs. The correlation of *z* scores between MDD and recurrent MDD was high, but not equal to 1 (*r* = .77, *p* < .001), indicating that there are both shared and trait-specific variants with sex-differentiated effects for these two overlapping definitions of MDD, although it should be noted that subtle differences in population structure could also impact these results. Furthermore, we observed cross-trait sharing of sex-dependent genetic effects between ASD and ADHD as well as BD and schizophrenia, to name examples.

### Gene Set Enrichment Analysis of Genes With Sex-Differentiated Effects Across Traits

To investigate the biological function of the genes harboring SNPs with sex-differentiated genetic effects, we selected the top 0.1% of genes from each trait (Table S9 in Supplement 2), resulting in 346 genes that were mapped for gene set enrichment analysis. The top 100 gene sets enriched for genes with sex-differentiated effects are listed in Table S10 in Supplement 2. The gene sets enriched for sex-differentiated effects included neurogenesis, regulation of nervous system development, regulation of neuron differentiation, neuron differentiation, positive regulation of nervous system development, regulation of neuron projection development, and neuron development, among others.

### Between-Trait, Within-Sex Genetic Correlation Analysis

**Figure 3:**
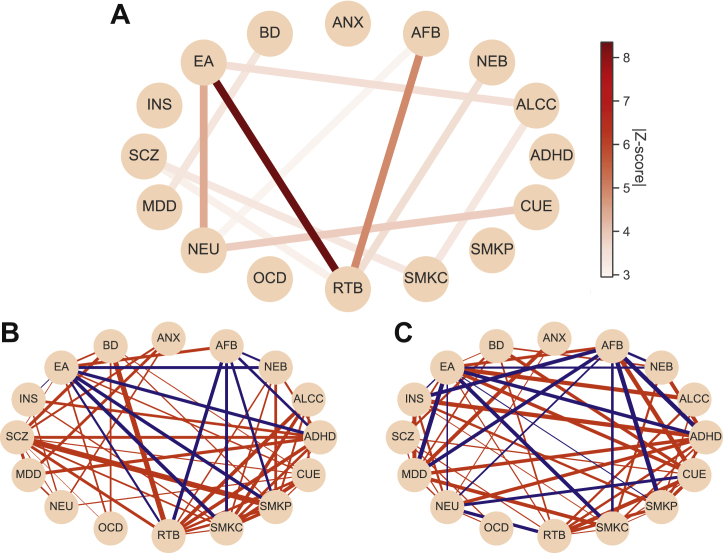
**(A)** Network plot showing between-trait genetic correlations with a significant sex difference as computed by *z* score. The edge color represents the absolute value of the *z* score for the difference in genetic correlation between the same 2 phenotypes in females vs. males. Only pairs of traits with false discovery rate corrected *q* < .05 sex difference are shown. **(B, C)** Between-trait, within-sex genetic correlation analysis. Network plots for genetic correlation estimates (*r_g_*) for pairs of traits in **(B)** males and **(C)** females, where each node represents a trait, and the edge represents positive (red) or negative (blue) genetic correlation. The thickness of the edge represents −log_10_(*q* value) of correlation significance. Only genetic correlations with false discovery rate corrected *q* < .05 are shown. Genetic correlations were visualized using the Python package Networkx (50) and Matplotlib (51). ADHD, attention-deficit/hyperactivity disorder; AFB, age at first birth; ALCC, alcohol use; ANX, anxiety disorders; ASD, autism spectrum disorder; BD, bipolar disorder; CUE, cannabis use (ever); EA, educational attainment; INS, insomnia; MDD, major depressive disorder; NEB, number of children ever born; NEU, neuroticism; OCD, obsessive-compulsive disorder; RTB, risk-taking behavior; SCZ, schizophrenia; SMKC, smoking (current); SMKP, smoking (previous).

The within-sex, between-trait genetic correlation results are presented as network plots (Figure 3A–C) and heatmaps (Figure S6 in Supplement 1). Most between-trait genetic correlations were not significantly different between males and females (Figure 3B, C). We detected several significant sex differences in the between-trait genetic correlations; see Table 2 and Figure 3A for top results and Table S11 in Supplement 2 for details. For example, educational attainment and risk-taking behavior were positively correlated in females but negatively correlated in males. Lifetime cannabis use and neuroticism were negatively correlated in females but positively correlated in males. The magnitude of *r_g_* was significantly greater in females than in males for a number of traits (e.g., risk-taking behavior and schizophrenia) and significantly smaller in females than in males for several trait pairs (e.g., number of children born and risk-taking behavior). Finally, we also observed trait pairs for which the estimated *r_g_* in one sex did not differ significantly from zero (Table S11 in Supplement 2), suggesting that either there was no significant genetic correlation between a given trait pair in one sex or the power to estimate this effect was too low.

**Table 2:** Top Results of Sex Differences in Cross-Trait Genetic Correlation Estimates

Trait 1	Trait 2	Females			Males			Sex Difference	
		*r*_*g*_	SE	*q* Value_*R*_	*r*_*g*_	SE	*q* Value_*R*_	*z* Score	*q* Value
EA	RTB	0.187	0.033	6.38 × 10^−8^	−0.144	0.033	4.29 × 10^−5^	−8.353	7.98 × 10^−15^
AFB	RTB	−0.035	0.046	.52	−0.344	0.054	1.23 × 10^−9^	−4.906	5.58 × 10^−5^
EA	NEU	−0.22	0.029	1.72 × 10^−13^	−0.064	0.029	.051	4.421	3.94 × 10^−4^
CUE	NEU	−0.142	0.055	.022	0.124	0.054	.044	3.866	3.32 × 10^−3^
NEB	RTB	0.116	0.063	.12	0.413	0.074	1.43 × 10^−7^	3.582	8.19 × 10^−3^
ALCC	EA	0.276	0.047	2.52 × 10^−8^	0.043	0.049	.47	−3.53	8.30 × 10^−3^
SCZ	SMKC	0.034	0.045	.52	0.214	0.046	1.54 × 10^−5^	3.301	.013
ALCC	SMKC	0.013	0.058	.86	0.292	0.069	8.97 × 10^−5^	3.326	.013
BD	MDD	0.565	0.079	4.95 × 10^−12^	0.057	0.142	.74	−3.367	.013
RTB	SCZ	0.326	0.043	3.13 × 10^−13^	0.157	0.038	1.07 × 10^−4^	−3.088	.024
AFB	NEU	−0.173	0.037	1.44 × 10^−5^	−0.028	0.048	.63	2.95	.035

The *z* scores were calculated using equation 1.

AFB, age at first birth; ALCC, alcohol use; BD, bipolar disorder; CUE, cannabis use (ever); EA, educational attainment; MDD, major depressive disorder; NEB, number of children ever born; NEU, neuroticism; RTB, risk-taking behavior; SCZ, schizophrenia; SMKC, smoking (current).

## Discussion

We investigated sex differences in the genetic architecture of 20 neuropsychiatric and behavioral traits using sex-stratified autosomal GWAS summary statistics. We used 3 complementary approaches, including estimation of SNP-based heritability, genetic correlation, and heterogeneity analyses, to evaluate sex differences within traits and across trait pairs. In line with the small effect sizes of individual common variants contributing to neuropsychiatric and behavioral phenotypes (see studies referenced in Table 1), our results suggest that sex differences in the common autosomal genetic architecture of these phenotypes are also small and polygenic, indicating that larger samples will be needed to detect these differences at the individual variant level. A corollary of this conclusion is that the large sex differences in prevalence of many psychiatric conditions are not fully explained by genetic factors and are more likely due to environmental, social, and systems-level biological differences. We caution, however, that it would be a mistake to interpret from these conclusions that genetic factors are unimportant in understanding phenotypic sex differences. As observed, even when genetic differences are small and dispersed throughout the genome, quantification of such differences can provide insight into biological processes that may impact both sexes but may be more detectable in one sex. Furthermore, the interaction between genetic risk and gendered social environments is likely to be complex, and much more research is needed to understand the effect of their interplay on mental health traits. Even with these limitations and complexities, we identified a small number of significant sex differences, described below.

For most traits and cross-trait pairs, we detected no consistent evidence of sex differences in SNP-*h*^*2*^, and the genetic correlations between males and females were moderate to high (mostly *r*_*g*_ > 0.8). This is consistent with prior twin-based studies that report limited evidence for substantial sex differences in heritability (40,41). Equivalent heritability does not preclude the possibility of sex differences in genetic architecture. However, these findings together suggest that most common autosomal genetic effects on psychiatric phenotypes are shared across sexes.

The phenotypes that showed sex differences were among those with the largest available sample sizes, indicating that sample size impacts power to detect sex differences, and consequently, the lack of significant differences for a given phenotype may be due to limited power resulting from small sample sizes (Table S12 in Supplement 2). For example, a recent larger analysis of gene-by-sex interaction in schizophrenia, BD, and MDD revealed significant associations for schizophrenia and MDD (20). We found that some pairs of genetically correlated traits also share sex-differentiated associations (e.g., ASD and ADHD; BD and schizophrenia). Taken together, these findings suggest that sex differences in the genetic architecture of neuropsychiatric and behavioral traits exist but are small and polygenic. They further support the hypothesis that SNPs with sex-differentiated genetic effects for one trait are also likely to exhibit sex-differentiated effects in phenotypically associated traits (18,42). Moreover, we found that the set of genes with the most sex-differentiated effects across all traits is enriched (among other gene sets) for neurogenesis, neuron differentiation, and development of nervous system gene functions.

For two traits with well-powered GWAS data (educational attainment and risk-taking behavior), several interesting results emerged. Both traits demonstrated similar SNP-*h*^*2*^ in males and females, indicating that there was no appreciable difference in the overall contribution of genetic factors in each sex. Also, neither trait demonstrated an excess of variants with sex-differentiated effects, showing that (at current sample sizes) there were few detectable sex-differentiated genetic effects. However, while the genetic correlation between males and females was high [educational attainment: *r*_*g*_ = 0.92, SE = 0.02, as previously reported (29); risk-taking behavior: *r*_*g*_ = 0.81, SE = 0.04], it was significantly less than 1 for both traits. These two traits were positively genetically correlated in females (*r*_*g*_ = 0.19) but negatively correlated in males (*r*_*g*_ = −0.14). These results may be explained by a scenario in which a large number of SNPs exist with very small sex-differentiated effects, which we remain underpowered to detect at individual loci but can observe in analyses of cumulative sex differences. An alternative possibility is that there are sex differences in ascertainment and measurement [e.g., research participation rates (43), or male and female subjects interpret the question about being a risk-taker differently], thus resulting in analysis of slightly different traits in males and females. Sex differences in ascertainment can impact genetic discovery; although such biases do not impact estimation of genetic correlation (43), they could theoretically impact sex differences in cross-trait genetic correlation or differences in heritability. In general, ascertainment effects (e.g., recruitment and participation biases) and measurement issues (e.g., phenotyping biases) should be carefully considered in future genetic studies of sex differences, for example, by using cohorts that are not subject to ascertainment biases (e.g., iPSYCH) or employing methods to mitigate this bias, such as inverse-probability weighted regression (43). Many of the current GWASs of behavioral traits are based on data from the UK Biobank (which is a relatively older, healthier, and wealthier female-biased cohort relative to the overall UK population) (44), whereas the case-control neuropsychiatric traits are typically ascertained from clinical populations.

These observations have important implications for the future of sex differences research. Although the majority of genetic effects for neuropsychiatric and behavioral traits are similar for males and females, sex-differentiated genetic effects can be identified, and we have shown for the first time that a portion are shared across traits. Comprehensive discovery of these effects will require larger sample sizes than for detection of main effects because of reduced statistical power in assessing the interaction between sex and genotype. We expect that as sample sizes increase, sex differences will continue to emerge but will be small in magnitude, reflecting the polygenic architecture of the phenotypes. For traits that are genetically correlated, we expect to observe cross-trait sharing of a portion of sex-differentiated genetic effects, as we have reported here. Furthermore, the large sex differences in prevalence of psychiatric disorders are unlikely to be explained entirely by common autosomal genetic factors. Additional studies investigating the interaction between cumulative genetic effects (including nonautosomal and rare variation), sex-differentiated cellular environments (e.g., the impact of sex hormones on genome regulation), and gendered social environments will be needed.

### Limitations and Considerations

We focused on neuropsychiatric and behavioral traits with available sex-stratified autosomal GWAS summary statistics. The GWAS cohorts we analyzed consisted exclusively of individuals of European ancestry, and thus we are unable to assess the degree to which these results are applicable to other ancestries. It is essential that future GWASs analyze cohorts representing diverse ancestries for a more comprehensive and inclusive analysis of sex differences. Furthermore, lack of access to genotype-level data restricted our analyses to methods developed for summary statistics. This precluded testing some hypotheses, such as the possibility of sex-specific genetic liability thresholds, which is most directly tested by comparing the polygenic score distributions in male and female subjects (15). Additionally, ascertainment and participation bias may confound identification of true sex differences (43). Estimation of SNP-*h*^*2*^ relies on several important assumptions (e.g., regarding the underlying genetic architecture and number of causal variants) (29,30) and can be influenced by many factors (e.g., sex-specific population prevalences, sex-dependent ascertainment methods for cases and controls, different sample sizes in males and females) (45, 46, 47). Accurate estimation of sex-specific population prevalences is complex given potential sex differences in referral, with underdiagnosis in one sex [e.g., as seen for ADHD (48)]. To account for these issues, we used prevalence estimates from two different populations (Denmark and United States) and a second method (LDAK) to test for consistency of results under different assumptions. SNP-*h*^*2*^ estimates based on the two different population prevalence estimates were highly correlated, indicating that in the absence of sex-specific ascertainment biases varying substantially by country, results using prevalence rates based on other populations (e.g., United Kingdom, from where many of the study participants are drawn) would likely be consistent as well. There were substantial differences in estimation based on either LDSC or LDAK, likely owing to the different model assumptions related to genetic architecture; the biggest discrepancies were for the traits with the smallest sample sizes (Figure S3 in Supplement 1); the true SNP-*h*^*2*^ estimate is likely to fall in between these estimates. Furthermore, it is likely that some of the GWAS summary statistics may have included data from super-screened and unscreened control subjects, which may have biased upward the genetic correlation estimates (49). Clear best practices for sex-specific genetic analyses have not yet been established and are needed for future studies.

The most direct method to identify SNPs with sex-dependent effects is to perform a genotype-by-sex interaction test. However, this requires individual-level genotype data. A sex-stratified analysis followed by a difference test, such as the *z* score used here, is equivalent to a genotype-by-sex interaction test when there is no interaction between covariates (e.g., principal components, age) and the strata (e.g., male and female) and the trait variances are equivalent in the two strata (33). If those assumptions hold, our stratified analyses will be conservative. Conversely, if those assumptions are violated, our stratified analysis will be robust to those covariate interactions and differences in residual variances when evaluating whether the common variant effects are heterogeneous across sex. For example, we have previously shown that *p* values from a genotype-by-sex interaction test were highly correlated with *z* score *p* values from the sex-stratified analysis (autosomal SNPs *r* = .65, *p* < 2.2 × 10^−16^, X chromosome SNPs *r* = .71, *p* < 2.2 × 10^−16^) in analysis of obsessive-compulsive disorder (22). However, subsequent systematic analysis of larger cohorts may illuminate whether these assumptions are violated and their impact on the interpretation of variants with sex-differentiated effects.

Another important limitation of our study is that we assessed only autosomal genetic effects, as summary statistics from the sex chromosomes were not available for the traits we analyzed. The sex chromosomes are frequently excluded from GWASs, owing to special consideration required for quality control and analyses, with many methods not allowing for the inclusion of sex chromosomes.

### Conclusions

Through within- and between-trait analyses, we find preliminary and modest evidence of sex-dependent autosomal genetic effects, with no single SNP exhibiting significant sex-differentiated genetic effects across neuropsychiatric and behavioral phenotypes among cohorts of European ancestry. However, consistent with the observed effect sizes of discovery GWASs of these phenotypes, these effects are small and polygenic, and therefore larger samples are needed to comprehensively identify these effects and characterize their functional contribution to complex traits. Furthermore, studies of sex differences taking into account nonautosomal and rare genetic variants as well as environmental (e.g., endogenous hormonal influences and exogenous exposures due to one’s sex), ethnic, and cultural differences are needed.

## Supplementary Material

Supplement 2

Key Resources Table

## References

[1] Westergaard D., Moseley P., Sørup F.K.H., Baldi P., Brunak S. (2019). Population-wide analysis of differences in disease progression patterns in men and women. Nat Commun.

[2] Traglia M., Bseiso D., Gusev A., Adviento B., Park D.S., Mefford J.A. (2017). Genetic mechanisms leading to sex differences across common diseases and anthropometric traits. Genetics.

[3] Rawlik K., Canela-Xandri O., Tenesa A. (2016). Evidence for sex-specific genetic architectures across a spectrum of human complex traits. Genome Biol.

[4] Khramtsova E.A., Davis L.K., Stranger B.E. (2019). The role of sex in the genomics of human complex traits. Nat Rev Genet.

[5] Naqvi S., Godfrey A.K., Hughes J.F., Goodheart M.L., Mitchell R.N., Page D.C. (2019). Conservation, acquisition, and functional impact of sex-biased gene expression in mammals. Science.

[6] Eaton N.R., Keyes K.M., Krueger R.F., Balsis S., Skodol A.E., Markon K.E. (2012). An invariant dimensional liability model of gender differences in mental disorder prevalence: Evidence from a national sample. J Abnorm Psychol.

[7] Plana-Ripoll O., Pedersen C.B., Holtz Y., Benros M.E., Dalsgaard S., de Jonge P. (2019). Exploring comorbidity within mental disorders among a Danish national population. JAMA Psychiatry.

[8] Rutter M., Caspi A., Moffitt T.E. (2003). Using sex differences in psychopathology to study causal mechanisms: Unifying issues and research strategies. J Child Psychol Psychiatry.

[9] Goldstein J.M., Holsen L., Huang G., Hammond B.D., James-Todd T., Cherkerzian S. (2016). Prenatal stress-immune programming of sex differences in comorbidity of depression and obesity/metabolic syndrome. Dialogues Clin Neurosci.

[10] Goldstein J.M., Hale T., Foster S.L., Tobet S.A., Handa R.J. (2019). Sex differences in major depression and comorbidity of cardiometabolic disorders: Impact of prenatal stress and immune exposures. Neuropsychopharmacology.

[11] Mareckova K., Holsen L.M., Admon R., Makris N., Seidman L., Buka S. (2016). Brain activity and connectivity in response to negative affective stimuli: Impact of dysphoric mood and sex across diagnoses. Hum Brain Mapp.

[12] Cross-Disorder Group of the Psychiatric Genomics Consortium (2019). Genomic relationships, novel loci, and pleiotropic mechanisms across eight psychiatric disorders. Cell.

[13] Anttila V., Bulik-Sullivan B., Finucane H.K., Walters R.K., Bras J., Brainstorm Consortium (2018). Analysis of shared heritability in common disorders of the brain. Science.

[14] Bulik-Sullivan B.K., Finucane H.K., Anttila V., Gusev A., Day F.R., Loh P.R. (2015). An atlas of genetic correlations across human diseases and traits. Nat Genet.

[15] Martin J., Walters R.K., Demontis D., Mattheisen M., Lee S.H., Robinson E. (2018). A genetic investigation of sex bias in the prevalence of attention-deficit/hyperactivity disorder. Biol Psychiatry.

[16] Walters R.K., Polimanti R., Johnson E.C., McClintick J.N., Adams M.J., Adkins A.E. (2018). Transancestral GWAS of alcohol dependence reveals common genetic underpinnings with psychiatric disorders. Nat Neurosci.

[17] Neale L. Rapid GWAS of thousands of phenotypes for 337,000 samples in the UK Biobank. http://www.nealelab.is/blog/2017/7/19/rapid-gwas-of-thousands-of-phenotypes-for-337000-samples-in-the-uk-biobank.

[18] Mitra I., Tsang K., Ladd-Acosta C., Croen L.A., Aldinger K.A., Hendren R.L. (2016). Pleiotropic mechanisms indicated for sex differences in autism. PLoS Genet.

[19] Grove J., Ripke S., Als T.D., Mattheisen M., Walters R.K., Won H. (2019). Identification of common genetic risk variants for autism spectrum disorder. Nat Genet.

[20] Blokland G.A.M., Grove J., Chen C.Y., Cotsapas C., Tobet S., Handa R. (2020). Sex-dependent shared and non-shared genetic architecture across mood and psychotic disorders. bioRxiv.

[21] Hammerschlag A.R., Stringer S., de Leeuw C.A., Sniekers S., Taskesen E., Watanabe K. (2017). Genome-wide association analysis of insomnia complaints identifies risk genes and genetic overlap with psychiatric and metabolic traits. Nat Genet.

[22] Khramtsova E.A., Heldman R., Derks E.M., Davis L.K., Stranger B.E., Tourette Syndrome/Obsessive-Compulsive Disorder Working Group of the Psychiatric Genomics Consortium (2019). Sex differences in the genetic architecture of obsessive-compulsive disorder. Am J Med Genet B Neuropsychiatr Genet.

[23] Duncan L.E., Ratanatharathorn A., Aiello A.E., Almli L.M., Amstadter A.B., Ashley-Koch A.E. (2018). Largest GWAS of PTSD (N=20 070) yields genetic overlap with schizophrenia and sex differences in heritability. Mol Psychiatry.

[24] Strawbridge R.J., Ward J., Lyall L.M., Tunbridge E.M., Cullen B., Graham N. (2018). Genetics of self-reported risk-taking behaviour, trans-ethnic consistency and relevance to brain gene expression. Transl Psychiatry.

[25] Clarke T.K., Adams M.J., Davies G., Howard D.M., Hall L.S., Padmanabhan S. (2017). Genome-wide association study of alcohol consumption and genetic overlap with other health-related traits in UK Biobank (N=112 117). Mol Psychiatry.

[26] Schumann G., Liu C., O’Reilly P., Gao H., Song P., Xu B. (2016). KLB is associated with alcohol drinking, and its gene product β-Klotho is necessary for FGF21 regulation of alcohol preference. Proc Natl Acad Sci U S A.

[27] Barban N., Jansen R., de Vlaming R., Vaez A., Mandemakers J.J., Tropf F.C. (2016). Genome-wide analysis identifies 12 loci influencing human reproductive behavior. Nat Genet.

[28] Okbay A., Beauchamp J.P., Fontana M.A., Lee J.J., Pers T.H., Rietveld C.A. (2016). Genome-wide association study identifies 74 loci associated with educational attainment. Nature.

[29] Hübel C., Gaspar H.A., Coleman J.R.I., Finucane H., Purves K.L., Hanscombe K.B. (2019). Genomics of body fat percentage may contribute to sex bias in anorexia nervosa. Am J Med Genet B Neuropsychiatr Genet.

[30] Bulik-Sullivan B.K., Loh P.R., Finucane H.K., Ripke S., Yang J., Schizophrenia Working Group of the Psychiatric Genomics Consortium (2015). LD Score regression distinguishes confounding from polygenicity in genome-wide association studies. Nat Genet.

[31] Speed D., Balding D.J. (2019). SumHer better estimates the SNP heritability of complex traits from summary statistics. Nat Genet.

[32] Roden D.M., Pulley J.M., Basford M.A., Bernard G.R., Clayton E.W., Balser J.R. (2008). Development of a large-scale de-identified DNA biobank to enable personalized medicine. Clin Pharmacol Ther.

[33] Pedersen C.B., Mors O., Bertelsen A., Waltoft B.L., Agerbo E., McGrath J.J. (2014). A comprehensive nationwide study of the incidence rate and lifetime risk for treated mental disorders. JAMA Psychiatry.

[34] Winkler T.W., Justice A.E., Cupples L.A., Kronenberg F., Kutalik Z., Heid I.M. (2017). Approaches to detect genetic effects that differ between two strata in genome-wide meta-analyses: Recommendations based on a systematic evaluation. PLoS One.

[35] Kunsch H.R. (1989). The jackknife and the bootstrap for general stationary observations. Ann Stat.

[36] Meijer E., Van Der Leeden R., Busing FMTA (1999). Delete-m Jackknife for Unequal m. Stat Comput.

[37] Watanabe K., Taskesen E., van Bochoven A., Posthuma D. (2017). Functional mapping and annotation of genetic associations with FUMA. Nat Commun.

[38] Watanabe K., Stringer S., Frei O., Mirkov M.U., Polderman T.J.C., van der Sluis S. (2019). A global view of pleiotropy and genetic architecture in complex traits. Nat Genet.

[39] Dalsgaard S., Thorsteinsson E., Trabjerg B.B., Schullehner J., Plana-Ripoll O., Brikell I. (2020). Incidence rates and cumulative incidences of the full spectrum of diagnosed mental disorders in childhood and adolescence. JAMA Psychiatry.

[40] Polderman T.J., Benyamin B., de Leeuw C.A., Sullivan P.F., van Bochoven A., Visscher P.M. (2015). Meta-analysis of the heritability of human traits based on fifty years of twin studies. Nat Genet.

[41] Bernabeu E., Canela-Xandri O., Rawlik K., Talenti A., Prendergast J., Tenesa A. (2020). Sexual differences in genetic architecture in UK Biobank. bioRxiv.

[42] Winkler T.W., Justice A.E., Graff M., Barata L., Feitosa M.F., Chu S. (2015). The influence of age and sex on genetic associations with adult body size and shape: A large-scale genome-wide interaction study. PLoS Genet.

[43] Pirastu N., Cordioli M., Nandakumar P., Mignogna G., Abdellaoui A., Hollis B. (2021). Genetic analyses identify widespread sex-differential participation bias [published online ahead of print Apr 22]. Nat Genet.

[44] Fry A., Littlejohns T.J., Sudlow C., Doherty N., Adamska L., Sprosen T. (2017). Comparison of sociodemographic and health-related characteristics of UK Biobank participants with those of the general population. Am J Epidemiol.

[45] van Rheenen W., Peyrot W.J., Schork A.J., Hong Lee S., Wray N.R. (2019). Genetic correlations of polygenic disease traits: From theory to practice. Nat Rev Genet.

[46] Schork A., Hougaard D., Nordentoft M., Mors O., Boerglum A., Mortensen P.B. (2019). Exploring contributors to variability in estimates of SNP-heritability and genetic correlations from the iPSYCH case-cohort and published meta-studies of major psychiatric disorders. bioRxiv.

[47] Trzaskowski M., Mehta D., Peyrot W.J., Hawkes D., Davies D., Howard D.M. (2019). Quantifying between-cohort and between-sex genetic heterogeneity in major depressive disorder. Am J Med Genet B Neuropsychiatr Genet.

[48] Staller J., Faraone S.V. (2006). Attention-deficit hyperactivity disorder in girls. CNS Drugs.

[49] Kendler K.S., Chatzinakos C., Bacanu S. (2020). The impact on estimations of genetic correlations by the use of super-normal, unscreened, and family-history screened controls in genome wide case-control studies. Genet Epidemiol.

[50] U.S. Department of Energy Office of Scientific and Technical Information Exploring network structure, dynamics, and function using NetworkX. https://www.osti.gov/biblio/960616.

[51] Hunter J.D. (2007). Matplotlib: A 2D graphics environment. Comput Sci Eng.

