# Supplementary material for: Examining Sex-Differentiated Genetic Effects Across Neuropsychiatric and Behavioral Traits
Source: Biol Psychiatry. 2021 Jun 15;89(12):1127–37. doi: 10.1016/j.biopsych.2020.12.024 (PMC8163257; doi:10.1016/j.biopsych.2020.12.024)
Supplement: Supplement 1 [file mmc1.pdf]

Examining Sex-Differentiated Genetic Effects Across Neuropsychiatric and Behavioral Traits

Supplement 1

Supplemental Text

Sex-stratified datasets

Sex-stratified genome-wide association study (GWAS) summary statistics for most traits were based on published data obtained by downloading from public repositories (e.g. http://www.med.unc.edu/pgc/results-and-downloads) or directly from analysts (see **Table 1** for details), with several exceptions, as described below.

PGC data for schizophrenia, major depressive disorder (MDD), and bipolar disorder were processed using a unified quality control pipeline as part of a separate study [1]. As part of this pipeline, samples were removed if they showed relatedness within or between datasets (PI-HAT <0.1). A subset of the MDD samples with recurrent MDD were also used for a sex-stratified analysis.

Unpublished sex-stratified GWAS summary-statistics for anxiety diagnosis, MDD diagnosis, and current and previous smoking were downloaded from http://www.nealelab.is/uk-biobank. The UK Biobank (UKBB) phenotype codes that were used are: “self-reported anxiety/panic attacks” (20002_1287), “self-reported depression” (20002_1286), “smoking status previous” (20116_1), and “smoking status current” (20116_2). Sex-stratified summary data from 2 other unpublished sex-stratified phenotypes in UK Biobank (neuroticism and cannabis use) were also available from co-authors; the UK Biobank codes that were used are: “neuroticism total score of 12 dichotomous items of the Eysenck Personality Questionnaire Revised Short Form” (20127) and "ever taken cannabis” (20453).

For ASD, 2 datasets were meta-analysed based on data from 2 publications [2,3] using an inverse-variance weighted fixed effects model, implemented in METAL [4]; the genetic correlation (in LDSC [5]) between these 2 ASD datasets was: rg(SE)=0.70(0.11) in males and could not be estimated in females due to low sample size.

For 2 phenotypes, data were available from 2 different sources and we assessed the genetic correlations between them and used only one of the datasets going forward; for MDD, data were available from the PGC and the UK Biobank (LDSC rg(SE)=1.16(0.37) for males & 0.60(0.11) for females) and we selected the PGC data as it was based on a rigorously

phenotyped clinical case-control dataset; for alcohol consumption data were available from 2 published studies [6,7] (rg(SE)=0.66(0.11) for males & 1.18(0.27) for females) and we used data from the larger study [6].

Quality control & processing datasets for analyses

The majority of available datasets had already undergone rigorous quality control, as described in each study publication (see **Table 1** for references and **Table S1** for data availability). We performed several additional steps, as follows. All datasets were on the same genomic build (hg19). SNPs were filtered for minor allele frequency (MAF), imputation quality, and sample size (N.B. different filters were applied for LDSC & meta-analyses; see below).

LDSC

The default parameters of LDSC (as per the script munge_sumstats.py) were used based on available columns provided in the shared datasets, removing variants as follows: MAF<=0.01, INFO<=0.9, N<(90th percentile N)/1.5, indels, strand-ambiguous SNPs and variants not in HapMap-3. Where the sample size was not provided per variant, the total sample size was provided to LDSC.

Sex-specific trait prevalences for estimating SNP-h^2

We obtained sex-specific trait prevalence estimates from the USA and cumulative incidence rates from Denmark, to compare the SNP-h^2 estimates using 2 different sources of information. The US-based estimates were derived from a hospital-based cohort of 752,436 patients who meet a medical home definition for Vanderbilt University Medical Center and whose de-identified electronic health record (EHR) is in the clinical research database [8]; see **Tables S2-S3** for details. The medical home is a heuristic definition aimed at reducing the influence of missing data. The medical home definition restricts the total sample to a subpopulation with at least 5 codes over a period of at least 3 years. This filters individuals who may have visited Vanderbilt for only a small number of specialty visits, but who receive their regular primary care in a community care setting. The requirement of a more complete medical record facilitates prevalence estimations for common mental health conditions seen typically in the primary care setting at Vanderbilt. We used estimates based on adults (age >18 years), except for ADHD and ASD, which included pediatric patients. To estimate prevalence of each phenotype (**Table S2**), we included individuals with at least one ICD code for each phenotype as the numerator, and 'medical home' hospital population as the denominator. However, hospital-based population prevalence estimates may be biased due to over-representation of individuals with more severe health-related conditions and higher levels of comorbidity.

Additionally, these prevalence estimates may not generalize to populations outside of the USA. Therefore, we also used sex-specific cumulative incidence rates at age 50 years, based on individuals identified through inpatient and outpatient care in Denmark [9], as well as childhood-specific (age <18 years) estimates for the 2 neurodevelopmental disorders in our analyses (ADHD and ASD), based on a more recent Danish study, as the discovery GWAS for these traits were based on samples of mostly children [10]; see **Table S2**.

Gene-based analysis, differential gene expression, and gene-set enrichment analysis of genes with sex-differentiated effects

We used the Functional Mapping and Annotation of GWAS (FUMA) SNP2GENE web tool [11], to perform gene-based analysis using MAGMA v1.08 [12,13], where SNPs are mapped to genes if they fall within the gene start and end sites (the default window size of 0kb) based on the 1000 Genomes project reference panel. Z-scores and their p-values computed for each trait were used as an input. We have applied the mean gene analysis model, a test of weighted mean SNP association. In this analysis, the mean of the chi^2 statistic for the SNPs in a gene is calculated and the p-value is obtained from a known approximation of the sampling distribution [14,15]. The genome-wide significance threshold is defined as 0.05/number of genes to which the SNPs are mapped. The number of genes that is analyzed through MAGMA depends on the SNPs represented in the summary statistics files. As shown in Table S7 the output of MAGMA results in an average ~18k genes. Some genes may be missed in the analysis if SNPs are not represented in a particular GWAS.

We examined whether the genes with genome-wide significant difference (from MAGMA analysis) between males and females with trait association demonstrate differential by sex expression in brain tissues from the Genotype Tissue Expression project v8 (https://www.gtexportal.org/home/datasets) [16].

After mapping SNPs to genes, we filtered the top 0.1% (with the lowest $-\log_{10}$(p-value)) from the gene-based MAGMA analysis, as genes with sex-differentiated effects and combined these sets across phenotypes. We selected the 0.1% threshold to test a set of genes with the greatest sex difference in effects, that did not exceed ~2000 genes, which is the input gene set limit for the Gene Set Enrichment Analysis (GSEA) tool (https://software.broadinstitute.org/gsea/index.jsp). We also evaluated the top 0.5% of genes to test sensitivity to this threshold. We observed that 57 out of the top 100 gene sets overlap between the 0.1% and 0.5% top genes which went into the GSEA. Finally, we computed a gene set overlap analysis using GSEA on the combined set of genes with sex-differentiated effects with collection C5 (GO biological process, GO cellular component, and GO molecular

functions) from MSigDB to investigate which functions may contribute to phenotypic differences observed for the traits.

We chose to calculate per-SNP sex difference z-scores and then used them for gene-based and gene set analyses. An alternative approach would be to determine genes and gene sets separately in males and females and then examine the overlap. Given the sample sizes of the sex-stratified GWAS that are currently available to the scientific community for neuropsychiatric and behavioral traits, we have low power to detect genome-wide significant associations within each sex for many of the phenotypes examined. Thus, we would have to set a threshold for top genes arbitrarily. If this is done based on the p-value, the sample size difference between the sexes would bias the number of significant variants/genes observed at that threshold. For this reason we decided to compute z-scores based on effect betas and standard errors to determine sex-biased SNP- and gene-level associations. Additionally, given our approach to focus on genes across phenotypes, setting a single p-value threshold would not be appropriate. Instead we decided to focus on the top 0.1% and 0.5% of the genes for each trait. Additionally, the added value of the z-score approach is that it allowed us to look at the genes with the largest difference between the sexes rather than analyzing a mix of shared and unique genes and utilizing a mix of those genes in the gene-set enrichment analyses.

Power calculations

We examined the range of power to detect differences from 1 in r_g estimates between males and females for a given trait, using previously described equations modified to test for differences from 1 [17]. We did this for all traits included in the genetic correlation analyses (i.e. those with non-zero SNP-h^2 in both sexes). We summarise the assumptions and outcomes of these power calculations in **Table S12**. Given the available sample sizes and estimated sex-specific SNP-h^2, we determined that for case-control traits, we have >80% power to detect significant differences from r_g=1, ranging from good power to detect relatively small differences (r_g=0.90 for schizophrenia) down to good power to detect only large deviation from 1 (r_g=0.36 for OCD). For quantitative traits, we have >80% power to detect significant differences for r_g=0.8 and above, for all traits. See **Table S12** for details.

Supplemental References

1. Blokland GAM, Grove J, Chen C-Y, Cotsapas C, Tobet S, Handa R, et al. Sex-Dependent Shared and Non-Shared Genetic Architecture Across Mood and Psychotic Disorders. bioRxiv. 2020; 2020.08.13.249813.

2. Mitra I, Tsang K, Ladd-Acosta C, Croen LA, Aldinger KA, Hendren RL, et al. Pleiotropic Mechanisms Indicated for Sex Differences in Autism. PLoS Genet. 2016;12: e1006425.

3. Grove J, Ripke S, Als TD, Mattheisen M, Walters RK, Won H, et al. Identification of common genetic risk variants for autism spectrum disorder. Nat Genet. 2019;51: 431–444.

4. Willer CJ, Li Y, Abecasis GR. METAL: fast and efficient meta-analysis of genomewide association scans. Bioinformatics. 2010;26: 2190–2191.

5. Bulik-Sullivan BK, Loh P-R, Finucane HK, Ripke S, Yang J, Schizophrenia Working Group of the Psychiatric Genomics Consortium, et al. LD Score regression distinguishes confounding from polygenicity in genome-wide association studies. Nat Genet. 2015;47: 291–295.

6. Clarke T-K, Adams MJ, Davies G, Howard DM, Hall LS, Padmanabhan S, et al. Genome-wide association study of alcohol consumption and genetic overlap with other health-related traits in UK Biobank (N=112 117). Mol Psychiatry. 2017;22: 1376–1384.

7. Schumann G, Liu C, O'Reilly P, Gao H, Song P, Xu B, et al. KLB is associated with alcohol drinking, and its gene product β-Klotho is necessary for FGF21 regulation of alcohol preference. Proceedings of the National Academy of Sciences. 2016. doi:10.1073/pnas.1611243113

8. Roden DM, Pulley JM, Basford MA, Bernard GR, Clayton EW, Balser JR, et al. Development of a Large-Scale De-Identified DNA Biobank to Enable Personalized Medicine. Clinical Pharmacology & Therapeutics. 2008. pp. 362–369. doi:10.1038/clpt.2008.89

9. Pedersen CB, Mors O, Bertelsen A, Waltoft BL, Agerbo E, McGrath JJ, et al. A comprehensive nationwide study of the incidence rate and lifetime risk for treated mental disorders. JAMA Psychiatry. 2014;71: 573–581.

10. Dalsgaard S, Thorsteinsson E, Trabjerg BB, Schullehner J, Plana-Ripoll O, Brikell I, et al. Incidence Rates and Cumulative Incidences of the Full Spectrum of Diagnosed Mental Disorders in Childhood and Adolescence. JAMA Psychiatry. 2019. doi:10.1001/jamapsychiatry.2019.3523

11. Watanabe K, Taskesen E, van Bochoven A, Posthuma D. Functional mapping and annotation of genetic associations with FUMA. Nat Commun. 2017;8: 1826.

12. de Leeuw CA, Mooij JM, Heskes T, Posthuma D. MAGMA: generalized gene-set analysis of GWAS data. PLoS Comput Biol. 2015;11: e1004219.

13. Leeuw C de, de Leeuw C, Sey NYA, Posthuma D, Won H. A response to Yurko et al: H-MAGMA, inheriting a shaky statistical foundation, yields excess false positives. bioRxiv. 2020. doi:10.1101/2020.09.25.310722

14. Brown MB. 400: A Method for Combining Non-Independent, One-Sided Tests of Significance. Biometrics. 1975;31: 987–992.

15. Hou C-D. A simple approximation for the distribution of the weighted combination of non-independent or independent probabilities. Stat Probab Lett. 2005;73: 179–187.

16. Oliva M, Muñoz-Aguirre M, Kim-Hellmuth S, Wucher V, Gewirtz ADH, Cotter DJ, et al. The impact of sex on gene expression across human tissues. Science. 2020;369. doi:10.1126/science.aba3066

17. Visscher PM, Hemani G, Vinkhuyzen AAE, Chen G-B, Lee SH, Wray NR, et al. Statistical Power to Detect Genetic (Co)Variance of Complex Traits Using SNP Data in Unrelated Samples. PLoS Genetics. 2014. p. e1004269. doi:10.1371/journal.pgen.1004269

Supplemental Figures

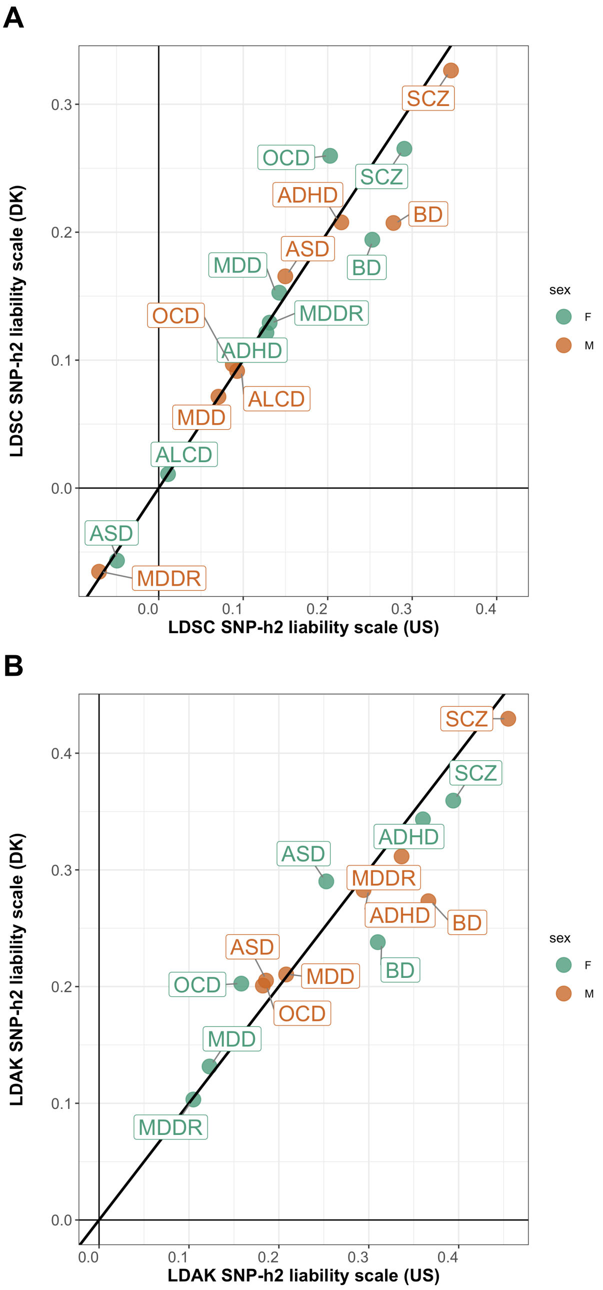

Figure S1. Scatter plots of the correlation of SNP-h^2 estimates using: US- and Denmark- (DK) based population prevalence rates, for 2 different SNP-h^2 estimation methods: (A) LDSC and (B) LDAK. Results show high correlation when using US-based (x-axis) and DK-based (y-axis) prevalence estimates for LDSC (r^2=0.97, p=4.7x10^{-10}) and LDAK (r^2=0.94, p=4.5x10^{-7}). Female SNP-h^2 estimates are shown in green and male SNP-h^2 estimates are shown in orange. The X:Y line is plotted in black. Standard errors (SE) are reported in the Tables S3 & S4. Phenotype abbreviations are listed in Table 1.

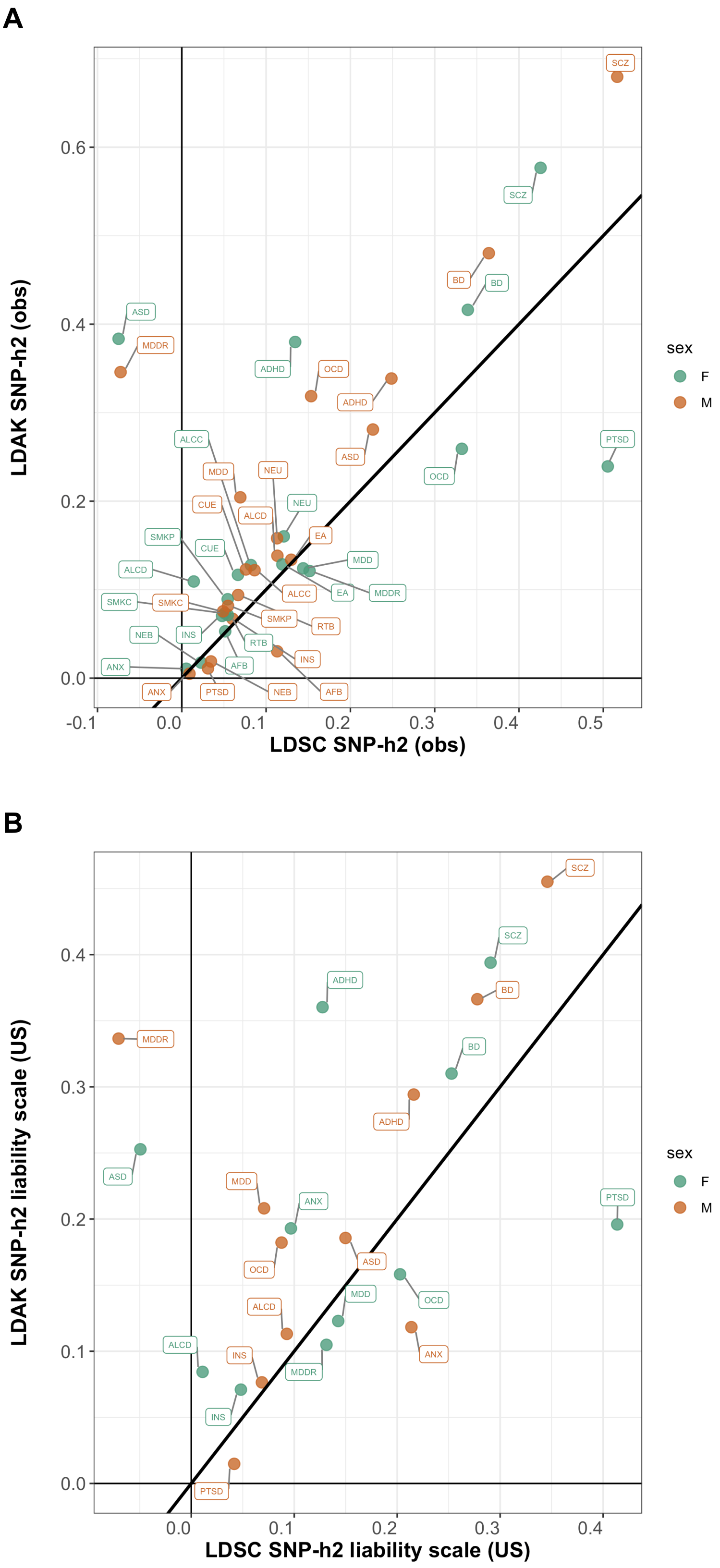
A
LDAK SNP-h2 (obs)
LDSC SNP-h2 (obs)
sex
F
M
SCZ
BD
ASD
MDDR
ADHD
OCD
PTSD
ALCC
MDD
NEU
CUE
ALCD
SMKP
EA
SMKC
RTB
NEB
INS
ANX
AFB
B
LDAK SNP-h2 liability scale (US)
LDSC SNP-h2 liability scale (US)

C

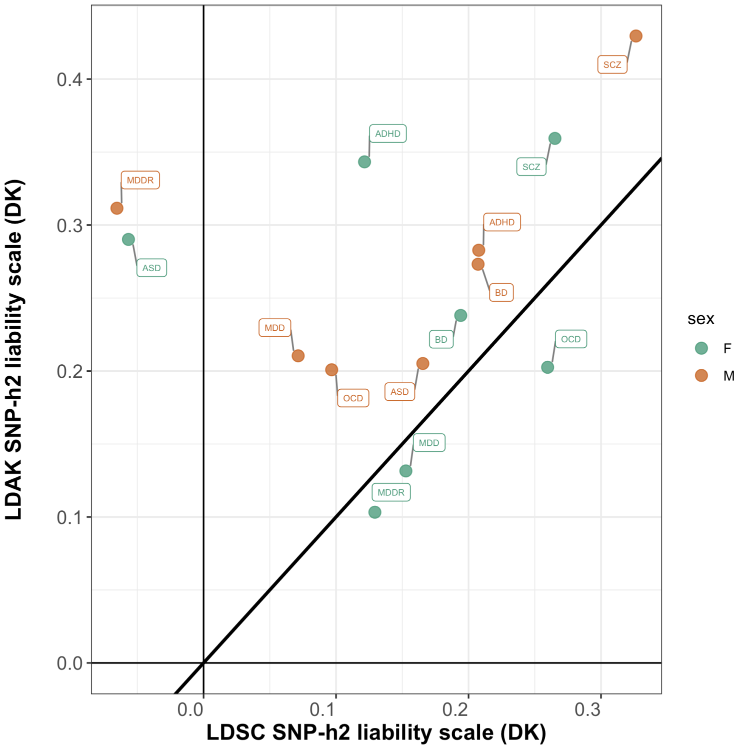

Figure S2. Correlation of SNP-h^2 estimates using LDSC and LDAK methods. Scatter plots of SNP-h^2 estimates on the (A) observed scale, (B) liability scale using US-based prevalence, and (C) liability scale using DK-based prevalence; each using LDSC (x-axis) and LDAK (y-axis). The X:Y line is plotted in black. Observed scale SNP-h^2 estimates from LDAK and LDSC methods are modestly correlated: (r=0.69, p=8.5x10^{-7} for all traits, r=0.85, p=3.3x10^{-11} excluding traits for which SNP-h^2 could not be reliably estimated in LDSC, i.e. PTSD & MDDR in males and ASD & ALCD in females. The liability scale SNP-h^2 estimates are only modestly correlated; US: r^2=0.44, p=0.042 for all traits, r^2=0.61, p=0.0077 excluding the 4 traits; DK: r^2=0.23, p=0.44 for all traits, r^2=0.61, p=0.035 excluding the 4 traits. Standard errors (SE) are reported in Tables S3 & S4. Phenotype abbreviations are listed in Table 1.

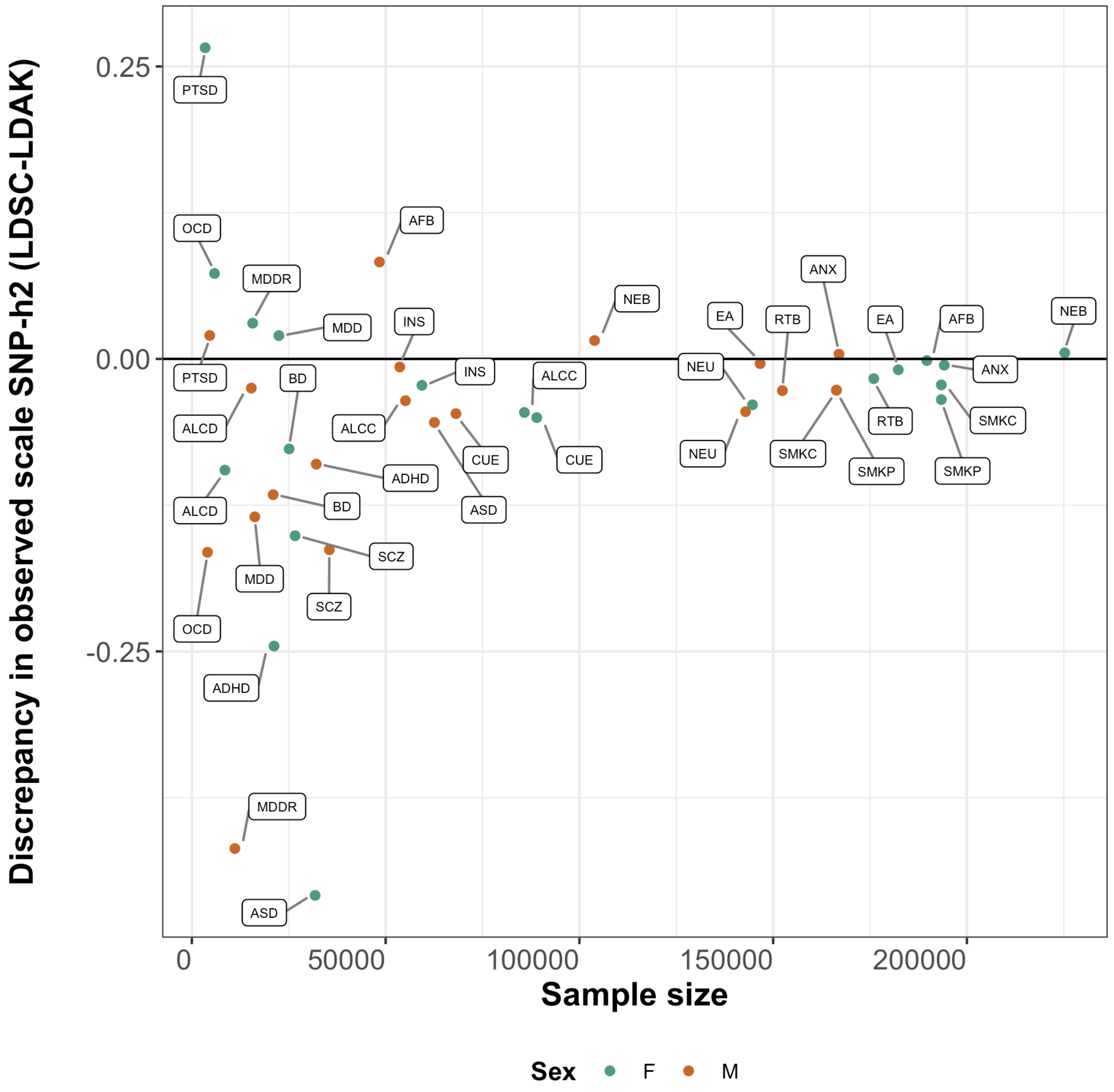

Figure S3. The effect of sample size on the difference between the LDSC and LDAK methods. Each point represents a value for the difference in SNP-h^2 estimated using two methods (LDSC and LDAK) for females (green) and males (orange). The sample size of the cohorts is shown on the x-axis, and the difference between SNP-h^2 estimates on the observed scale is shown on the y-axis. Phenotype abbreviations are listed in Table 1.

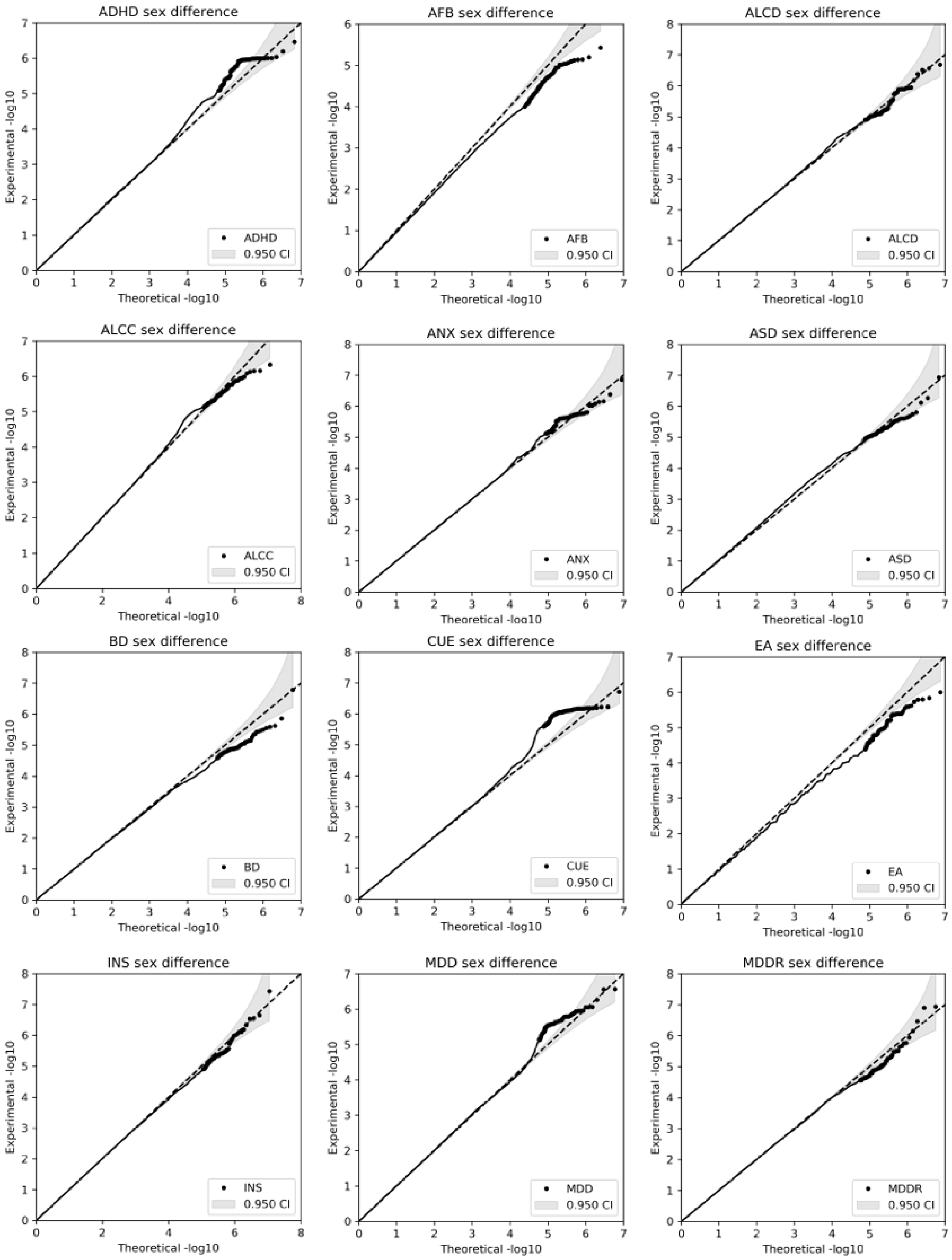
ADHD sex difference
AFB sex difference
ALCD sex difference
ALCC sex difference
ANX sex difference
ASD sex difference
BD sex difference
CUE sex difference
EA sex difference
INS sex difference
MDD sex difference
MDDR sex difference
Experimental -log10
Theoretical -log10
ADHD
AFB
ALCD
ALCC
ANX
ASD
BD
CUE
EA
INS
MDD
MDDR
0.950 CI

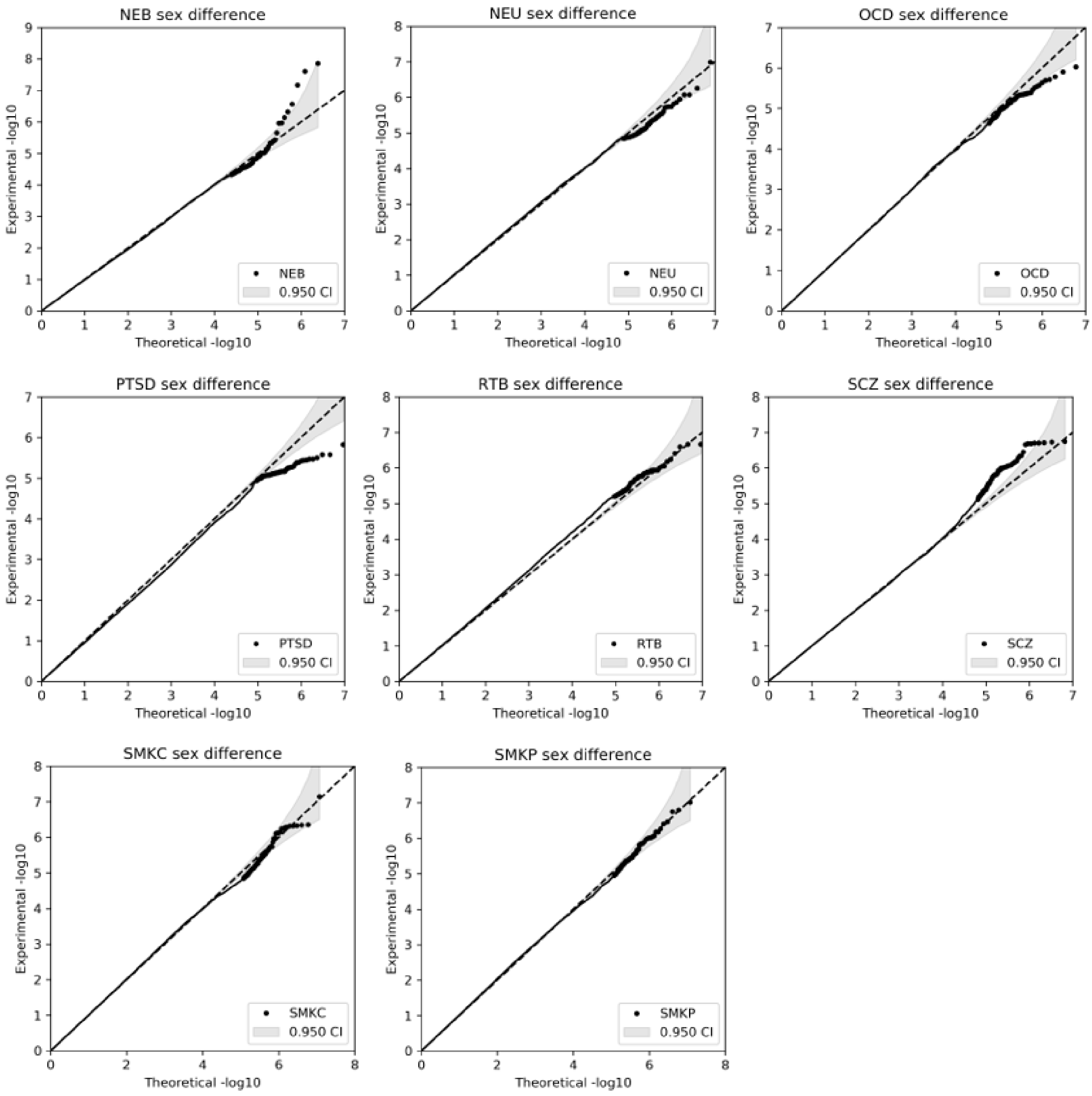

Figure S4. Quantile-quantile (QQ) plots of z-score p-values. Theoretical -log10(p-values) are shown on the x-axis, and experimental -log10(p-values) are shown on the y-axis. The X:Y line is plotted as a black dashed line and the grey area represents the 95% confidence interval. Phenotype abbreviations are listed in Table 1.

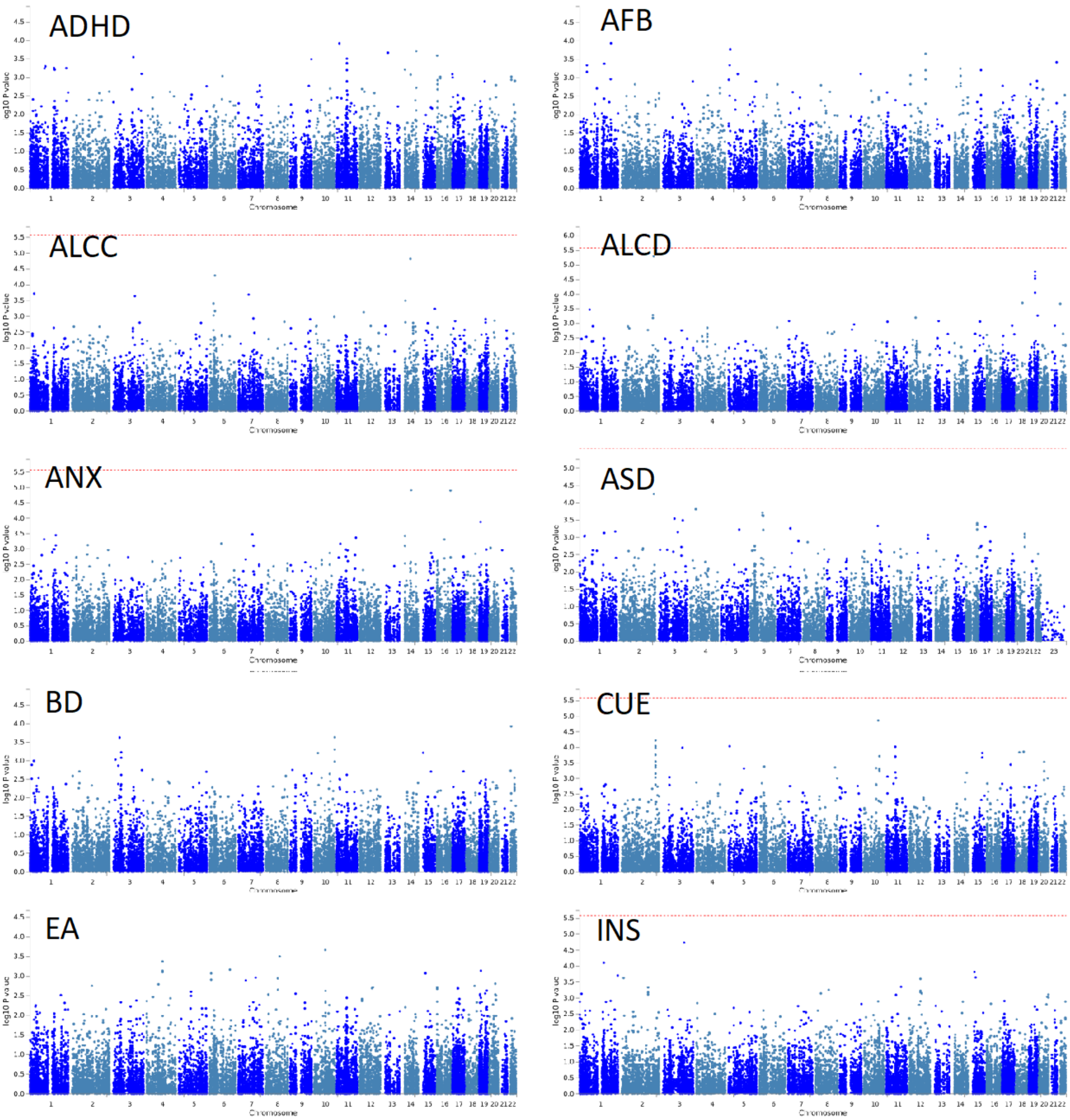
ADHD
AFB
ALCC
ALCD
ANX
ASD
BD
CUE
EA
INS
log10 P value
Chromosome

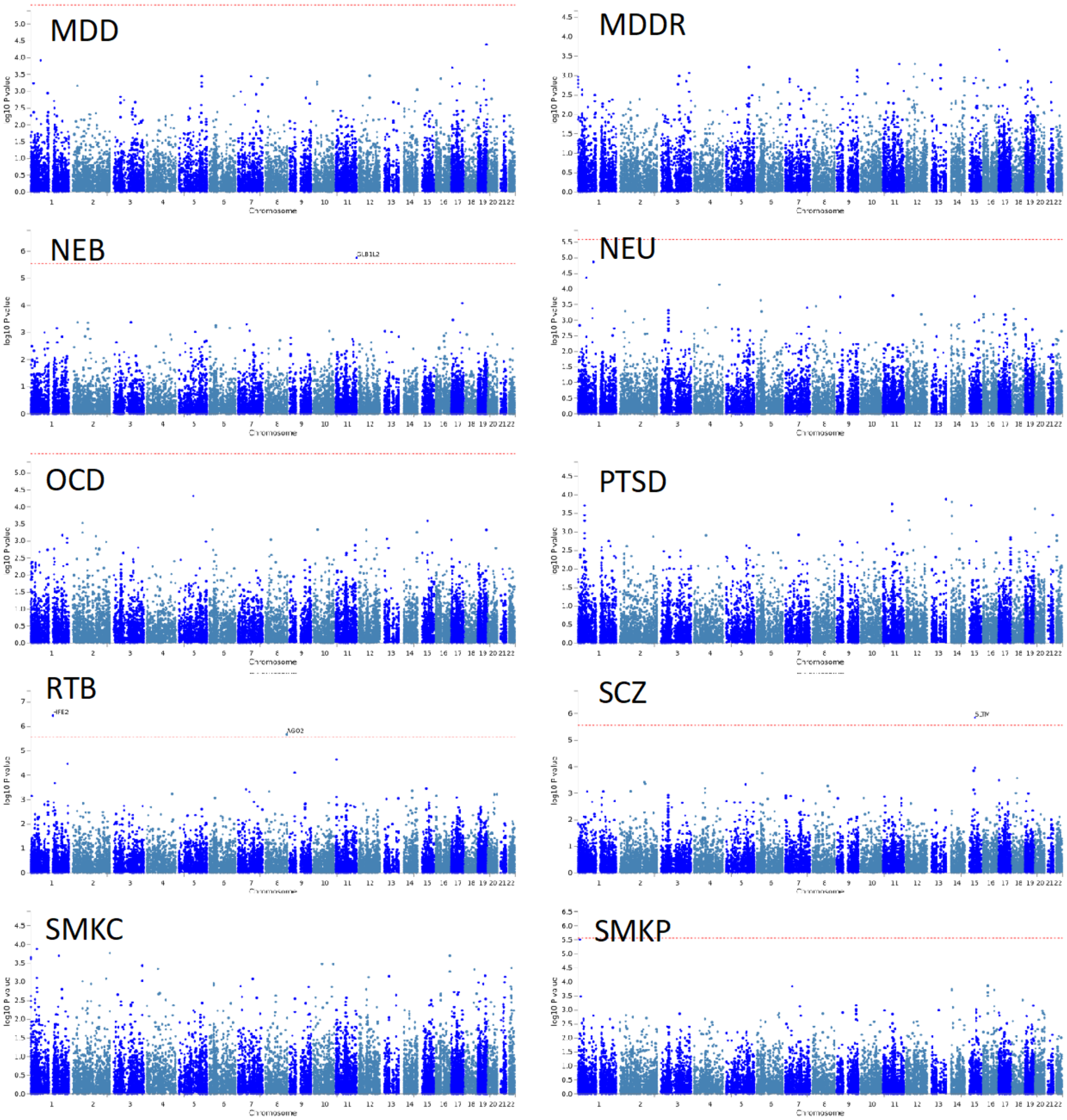

Figure S5. Manhattan plots of gene-based analysis in FUMA using z-score p-values. Each point represents a gene. Chromosome and base pair positions are shown on the x-axis, while the -log10(p-values) are shown on the y-axis. Phenotype abbreviations are listed in Table 1.

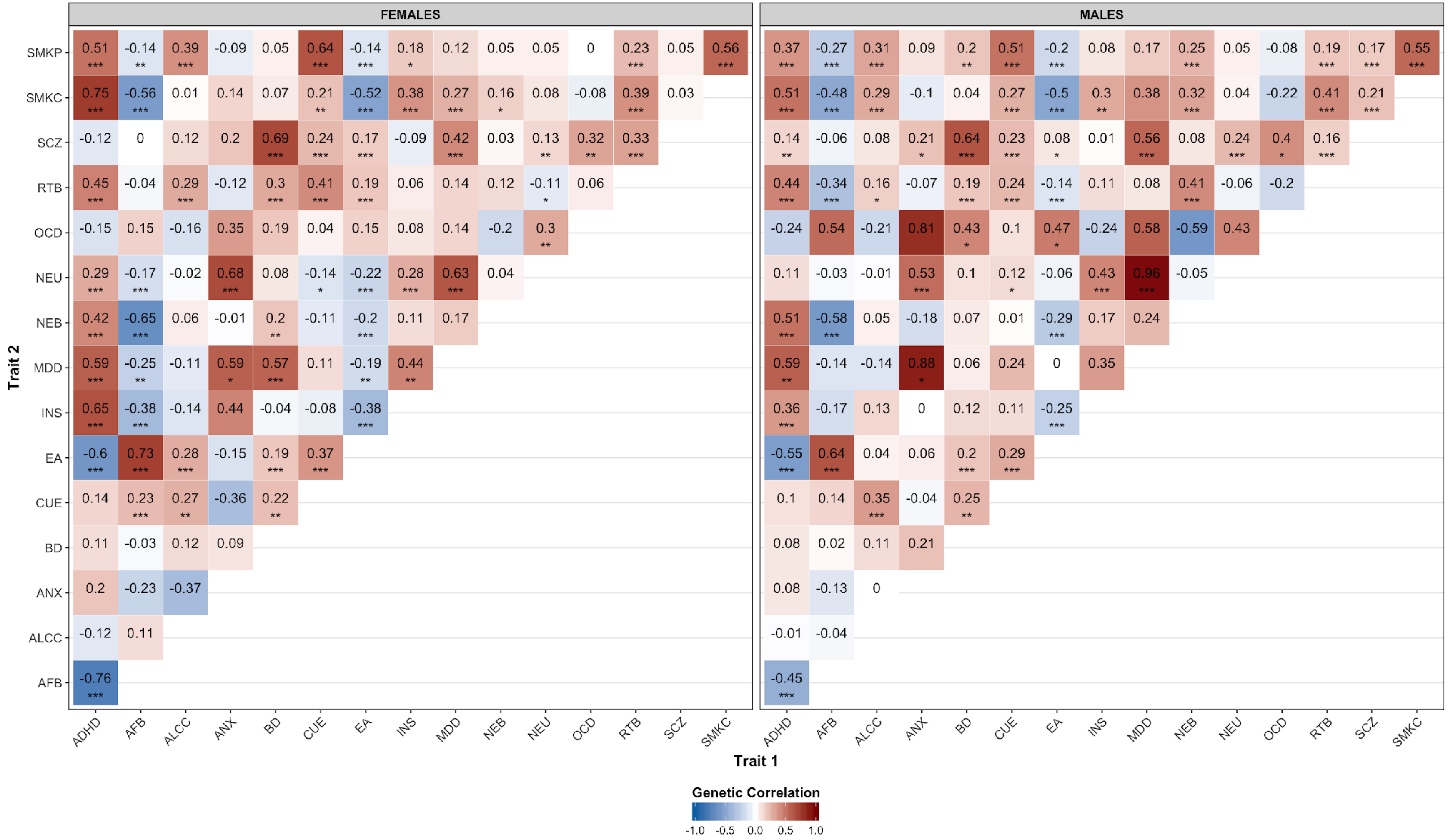

Figure S6. Heatmaps of genetic correlation analyses for females (left) and males (right). Red represents positive correlations and blue represents negative correlations. The correlation coefficient is listed in each cell. The significance of the correlations is denoted with asterisks, as follows: *p<0.05, **p<0.01, ***p<0.001. Phenotype abbreviations are listed in Table 1.
